# Anticholinesterase activity of *Areca Catechu*: *In Vitro* and *in silico* green synthesis approach in search for therapeutic agents against Alzheimer’s disease

**DOI:** 10.3389/fphar.2022.1044248

**Published:** 2022-11-04

**Authors:** Sushma Pradeep, Samudyata C. Prabhuswaminath, Pruthvish Reddy, Sudhanva M. Srinivasa, Ali A. Shati, Mohammad Y. Alfaifi, Serag Eldin I. Elbehairi, Raghu Ram Achar, Ekaterina Silina, Victor Stupin, Natalia Manturova, Daniel Glossman-Mitnik, Chandan Shivamallu, Shiva Prasad Kollur

**Affiliations:** ^1^ Department of Biotechnology and Bioinformatics, School of Life Sciences, JSS Academy of Higher Education and Research, Mysuru, Karnataka, India; ^2^ Department of Biotechnology, Acharya Institute of Technology, Bengaluru, Karnataka, India; ^3^ Adichunchanagiri Institute for Molecular Medicine, Adichunchanagiri University, Mandya, Karnataka, India; ^4^ Biology Department, Faculty of Sciences, King Khalid University, Abha, Saudi Arabia; ^5^ Cell Culture Lab, Egyptian Organization for Biological Products and Vaccines (VACSERA Holding Company), Agouza, Giza, Egypt; ^6^ Division of Biochemistry, School of Life Sciences, JSS Academy of Higher Education and Research, Mysuru, Karnataka, India; ^7^ Department of Surgery, Pirogov Russian National Research Medical University, Mascow, Russia; ^8^ Institute of Biodesign and Modeling of Complex Systems, I.M. Sechenov First Moscow State Medical University (Sechenov University), Moscow, Russia; ^9^ Laboratorio Virtual NANOCOSMOS, Departamento de Medio Ambiente y Energía, Centro de Investigación en Materiales Avanzados, Chihuahua, Chih, Mexico; ^10^ School of Physical Sciences, Amrita Vishwa Vidyapeetham, Mysuru, Karnataka, India

**Keywords:** Alzheheimer’s disease, anti-acetylcholinesterase and butyrylcholinesterase activity, neurofibrillary tangles, amyloid plaques, *Areca catechu* L., hydroxyapatite nanoparticles, molecular docking, Conceptual Density Functional Theory

## Abstract

For many years, the primary focus has been on finding effective treatments for Alzheimer’s disease (AD), which has led to the identification of promising therapeutic targets. The necessity for AD stage-dependent optimal settings necessitated a herbal therapy strategy. The plant species *Areca Catechu* L. (AC) was selected based on the traditional uses against CNS-related diseases. AC leaf extract were prepared using a Soxhlet extraction method and hydroxyapatite nanoparticles (HAp-NPs) were synthesized from the same (AC-HAp-NPs). Powder X-ray diffractometer (XRD), scanning electron microscopy (SEM), transmission electron microscopy (TEM), selected area electron diffraction (SAED) and fourier transform infrared spectroscopy (FTIR) were used to confirm the structure and morphology of the as-prepared AC-HAp-NPs. The crystalline character of the AC-HAp-NPs was visible in the XRD pattern. The synthesized material was found to be nanoflake, with an average diameter of 15–20 nm, according to SEM analysis. The TEM and SAED pictures also revealed the form and size of AC-HAp-NPs. *In vitro* anti-acetylcholinesterase and butyrylcholinesterase (AChE and BChE) activities of hydroxyapatite nanoparticles produced from an AC leaf extract was tested in this study. When compared to control, AC-HAp-NPs had higher anti-AChE and BChE activity. The anti-acetylcholinesterase action of phytoconstituents generated from AC leaf extract was mediated by 4AQD and 4EY7, according to a mechanistic study conducted utilizing *in silico* research. The global and local descriptors, which are the underpinnings of Conceptual Density Functional Theory (CDFT), have been predicted through the MN12SX/Def2TZVP/H_2_O model chemistry to help in the comprehension of the chemical reactivity properties of the five ligands considered in this study. The CDFT experiments are supplemented by the calculation of several useful calculated pharmacokinetics indices, their expected biological targets connected to the bioavailability of the five ligands in order to further the goal of studying their bioactivity.

## 1 Introduction

Alzheimer’s disease (AD) is a devastating neurological disease characterized by memory loss and cognitive impairment, which are the two most frequent symptoms Uddin et al. (2021). Due to the degeneration of cholinergic neurons in the basal forebrain, including senile plaques and neurofibrillary tangles, cognitive deficiency is consistent with the existence of cholinergic deficit in the neuropathological symptoms of Alzheimer’s disease (Sushma et al., 2020). Acetylcholine (ACh), the brain’s most essential natural neurotransmitter, is involved in memory formation, verbal and logical reasoning, and concentration. Both acetylcholinesterase (AChE) and butyrylcholinesterase (BChE) enzymes, on the other hand, significantly reduce ACh activity (Waring and Rosenberg., 2008). Inhibition of the cholinesterase enzymes (AChE and BChE) can ameliorate symptoms associated with the progressive loss of cholinergic function in AD by increasing ACh levels in various areas of the brain. Increased ACh concentration in the brain also promotes the expression of nicotinic ACh receptors linked to cognitive function, according to studies. This process could aid Alzheimer’s patients in forming new memories and recalling old ones. As a result of this “cholinergic hypothesis,” AChE and BChE inhibition has been recognized as the main therapeutic target (Atatreh et al., 2019).

Rather than targeting the etiological pathways, pharmacological treatment for AD focuses on treating symptoms and severity in advanced stages. The majority of the drugs now approved for AD are cholinesterase inhibitors, such as donepezil, rivastigmine, galantamine, and the NMDA antagonist memantine (Kim, 2018). Only donepezil, one of the most regularly prescribed AChE inhibitors, has been authorized for the treatment of all stages of AD. However, this medication causes a number of serious adverse effects, including GIT abnormalities, liver difficulties and gastrointestinal disturbances (Nistor et al., 2007). Given these constraints, it is worthwhile to seek out new lead compounds from a variety of sources, including plant-derived natural products. Natural compounds have shown to be promising sources of AChE inhibitors. Galantamine and rivastigmine, two currently approved medications for AD, are plant-derived alkaloids that provide symptomatic relief (Mahley et al., 2006).

In this regard, drug discovery research has focused on the design and development of therapeutic agents based on the numerous mechanisms implicated in AD. Although the design and synthesis of selective ligands pointing to single disease targets has traditionally been the key method in drug discovery research, due to the multifactorial pathophysiology of many diseases such as AD, this strategy has not always been fruitful. In this context, phytotherapy, which uses a combination of ingredients to treat a variety of ailments, is a novel and effective therapeutic option (Kametani and Hasegawa, 2018). *Areca Catechu* L. is a member of the Arecaceae family that can be found throughout Asia, the tropical Pacific, and parts of India. The plant’s leaves contains a variety of phytochemicals including as alkaloids, tannins, and polyphenols, and it has been shown to be effective in reducing the symptoms of AD (Martorana et al., 2010; Ferreira-Vieira et al., 2016). Our research aims to identify and compare possible candidates from the *A. catechu* seed for anticholinesterase activity in memory and cognitive function rejuvenation and improvement.

The goal of *in-silico* molecular docking (MD) approaches is to anticipate a ligand’s optimum binding mode to a macromolecular protein. It entails the creation of a variety of potential ligand conformations/orientations, or poses, within the protein binding site. Thus, a molecule target’s 3-D structure through X-ray crystallography/NMR or through computational approach based homology modeling forms a pre-requisite for MD. The molecular docking method utilizes several scoring standards to obtain the confirmation and affinity of the compound with the selected target. The best-docked pose of protein-drug complexes is subjected to molecular dynamics simulation (MDS), which is a computational technique for simulating the dynamic behaviour of molecular systems as a function of time, with all entities in the simulation box (ligands, proteins, and any explicit fluids) treated as flexible. In addition, protein-ligand complexes formed during the MDS were assessed to determine the types of binding free energies, which are used for the complex formation. Considering the anti-neuronal efficacy of the phytochemicals from *A. catechu*, 25 major phytochemicals were assessed against the major proteins responsible for causing AD, using the computational experiments listed above.

## 2 Materials and methods

The study’s entire chemical supply came from Loba Chemicals (Bangalore, India). Demineralized water was obtained using an ELGA RO system and used throughout the testing (Elga Veolia, Lane End, United Kingdom). Cu K (1.5406) radiation was used to record the crystalline phases using a Bruker X-ray diffractometer with a scan range of 20–70° and a scan rate of 2°/min (Bruker, Karlsruhe, Germany). The morphology and elemental composition of the samples, which were photographed using a Zeiss microscope, were examined using SEM and EDX mapping (Carl Zeiss, White Plains, NY, United States). TEM images and SAED patterns were obtained on a JEOL 2100F FEG apparatus operating at 200 kV after casting a drop of sample material for dispersion in ethanol on a Cu grid (JEOL, Akishima, Tokyo, Japan).

### 2.1 Plant material collection

Dr Shivlingaih performed the unambiguous authentication of the specimen that was then deposited in the herbarium with the voucher specimen number AC126 in the Department of Biotechnology and Bioinformatics, JSS AHER, Mysore, Karnataka, India. The *Areca Catechu* L. leaves material available in the university was collected and was further subjected to sterilization. To get rid of microscopic organisms and other dust particles, the leaves were first washed in single distilled water and 0.5% sodium hypochlorite solution, then in double distilled water. They were then shade dried for 45 days at room temperature (28.5°C). The dry components were then ground into a fine powder using a blender (Kollur et al., 2021).

### 2.2 Extraction of plant material and determination of phytochemicals

The Soxhlet apparatus was used to extract the powdered sample. 80 g of sample was placed in a thimble and extracted for 8 h with methanol as the solvent (24 cycles). The extracted materials were air-dried and kept at 4°C. It was also subjected to phytochemical analysis, both qualitative and quantitative, to determine the presence of different phytochemicals in the leaf extract (Pradeep et al., 2021). The produced extract was further utilized to prepare hydroxyapatite nanoparticles which intern was used to study the cholinesterase inhibitory activity.

### 2.3 Green synthesis of *Areca Catechu* derived hydroxyapatite nanoparticles

In order to synthesize HAp-NPs, leaf extract was used as the solvent to make 1M CaCl_2_ and 0.6M Na_2_HPO_4_, which were then each individually adjusted to pH 10.0 using 0.8M NaOH solution. A magnetic stirrer was used to vigorously agitate the CaCl_2_ solution at room temperature, and then Na_2_HPO_4_ solution was added drop by drop to produce a gelatinous precipitate. The following is a description of how HAp precipitates:

$$10{CaCl}_{2}+6{Na}_{2}{HPO}_{4}+8NaOH\to {Ca}_{10}{\left({PO}_{4}\right)}_{6}{\left(OH\right)}_{2}+20NaCl+6\;H_{2}O$$

The resulting precipitate was subjected to centrifugation to remove byproducts before being dried in a hot air oven for 6 h at 130°C to produce a dry cake that was ground into powder (Pradeep et al., 2021). To confirm the presence, formation, size and structure of nanoparticle the obtained HAp-NPs were further subjected to different characterization techniques such as SEM, EDX, TEM, XRD and FTIR.

### 2.4 Determination of cholinesterase (AChE/BChE) inhibitory assay

ChE inhibitory activity was determined using Ellman’s technique, which was previously described in a study. The enzyme hydrolyzes the substrate acetylthiocholine to create thiocholine, which interacts with Ellman’s reagent (DTNB) to yield 2-nitrobenzoate-5-mercapto-thiocholine and 5-thio-2-nitrobenzoate, which can be detected at 405 nm. In this assay, 25 µl of acetylthiocholine iodide (5 mM), 125 µl of DTNB (3 mM), 50 µl of buffer B (50 mM Tris-HCl, pH 8, 0.1 percent BSA), and test extract solution at different volume (20, 40, 80, 160 and 320 µl) or a negative control (25% DMSO in MeOH) were mixed and incubated for 10 min at 37°C. 25 µL of 0.05 U/mL AChE was added to start the reaction. At 405 nm, the absorbance was measured. BChE inhibition was determined in the same method as previously described, using 25 µL of 5 mM butyrylthiocholine iodide as a substrate and 0.05 U/ml of BChE as an enzyme. For both enzymes, galantamine was utilized as a positive control (Pradeep et al., 2021; Uddin et al., 2021).

### 2.5 *In silico* molecular docking studies

#### 2.5.1 Protein preparation, validation and active site prediction

**FIGURE 1:**
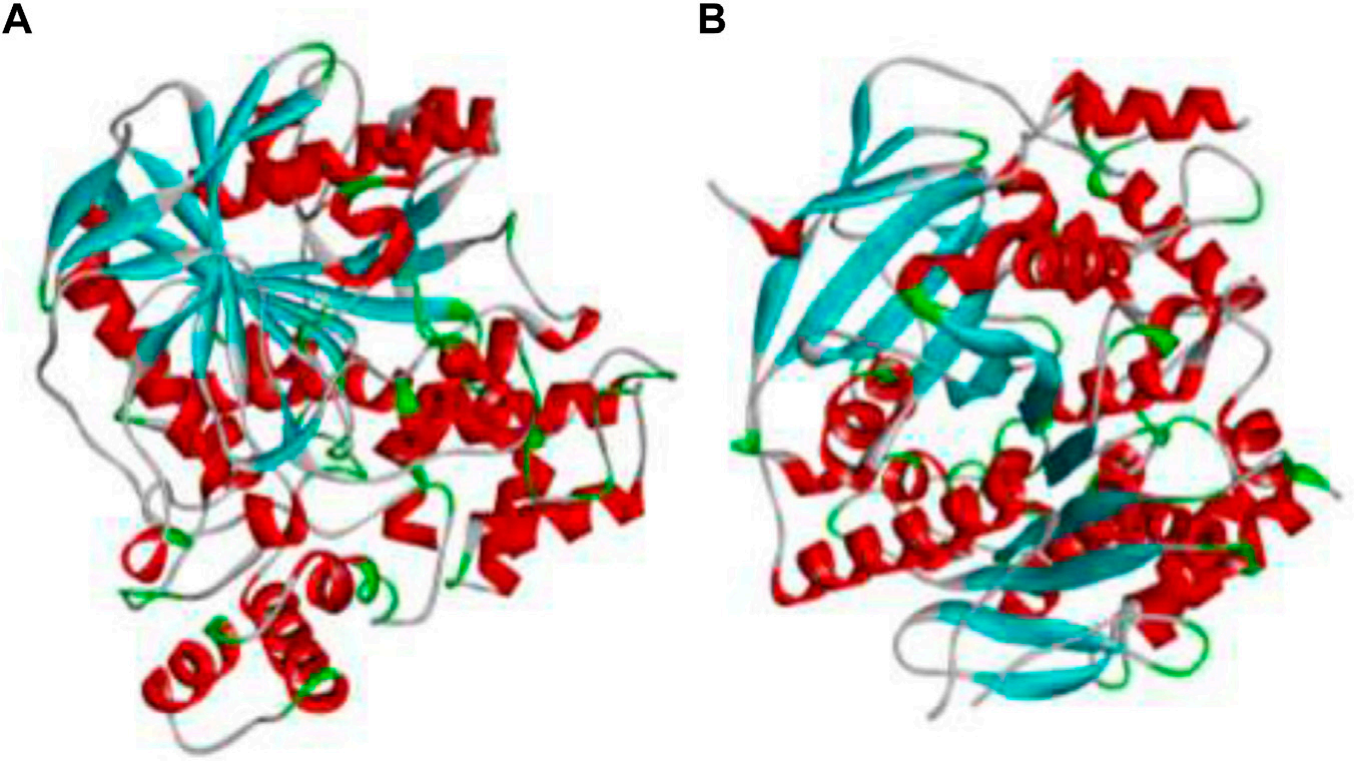
The 3D protein structures of **(A)** BChE (4AQD) and **(B)** AChE (4EY7) using Discovery Studio 3.1. visualization software.

The AChE and BChE are the main receptors that are majorly responsible in causing AD and their three dimensional structures were downloaded from Protein Data Bank with PDB IDs 4EY7 and 4AQD. These structures were visualized in Discovery Studio *3.1.* software, where the other small molecules already attached to the structures were removed and required amount of hydrogens were added, thus the resulting structures as shown in Figure 1 were saved and utilized for further *in silico* analysis (Pradeep et al., 2021).

An active site/catalytic site is a tiny area or cleft where the ligand molecule can attach to the receptor protein and create the desired result (Prasad et al., 2021). The identification of this active site residue in the target protein structure has numerous uses in molecular docking and new drug development. Due to the target protein’s frequent structural changes, identifying this catalytic binding site is difficult (Ankegowda et al., 2020).

#### 2.5.2 Lead molecule optimization

The IMPPAT (Indian Medicinal Plants, Phytochemistry and Therapeutics) database, which covers more than 1700 Indian medicinal plants with 1,100 therapeutic uses (https://cb.imsc.res.in/imppat/basicsearchauth), was used to search for the phytocompounds of Areca catechu leaf. According to their ADMET properties, the identified phytocompounds were retrieved, and the active compounds were checked for a number of parameters, including oral bioavailability (OB) 30%, blood-brain barrier (BBB), half-life (HL) 3 h, Lipinski’s rule of five, Caco-2 cells, drug-induced liver injury (DILI), clearance (CL) > 15 ml, molecular weight (MW), hydrogen bond donors (HbD) (PAINS).

Among which 25 compounds were selected for the *in silico* studies (Table 1). Their structures were drawn using Chemsketch software (Jain et al., 2021) and the geometry optimization and energy minimization of each molecule were carried out for all the structures using OpenBabel and Argus Lab software (Prasad K. S. et al., 2020).

**TABLE 1:** Representing the 2D images of all the 25 ligands and their binding energy details against 4AQD and 4EY7 proteins.

Ligand no	Compound/molecule name	Binding energy (kcal/mol)		Structure
		4AQD	4EY7	
1	Chlorogenic acid	−7.2	−9.6	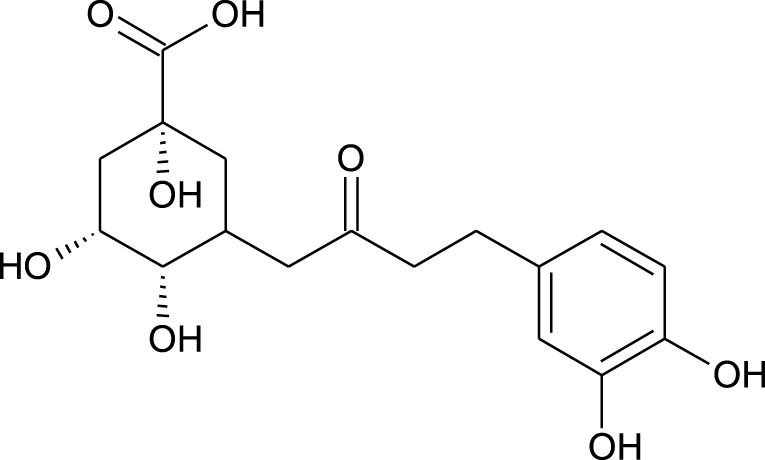
2	Lorartan Metabolite	-7.9	-11.3	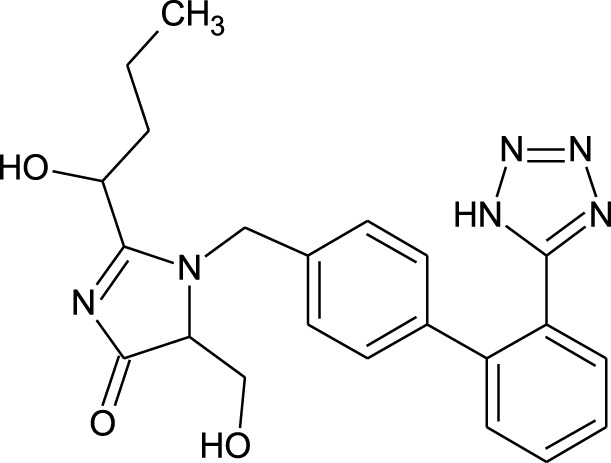
3	Gln Gln Gln	−6.7	−8.6	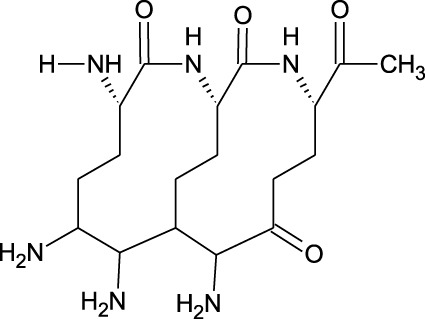
4	Dehydronimodipine	−10.1	−11.7	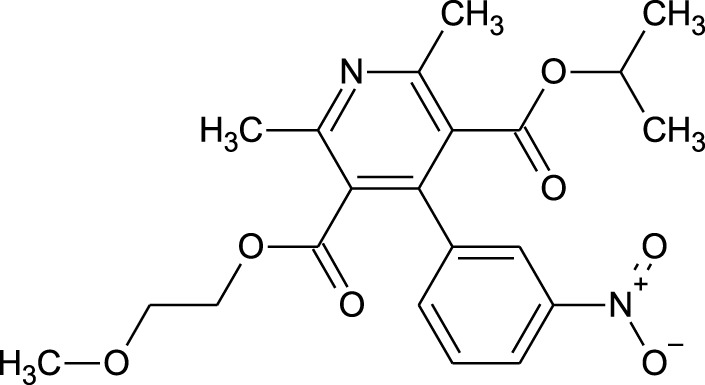
5	*N*-Acetylcilartatin	−6.7	−8.6	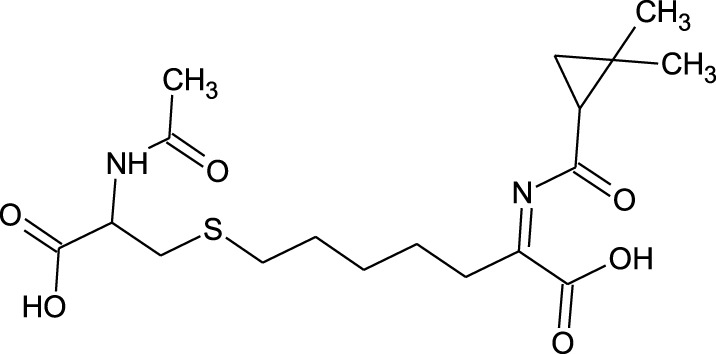
6	Sulindac sulfide glucuronide	−1.1	−1.1	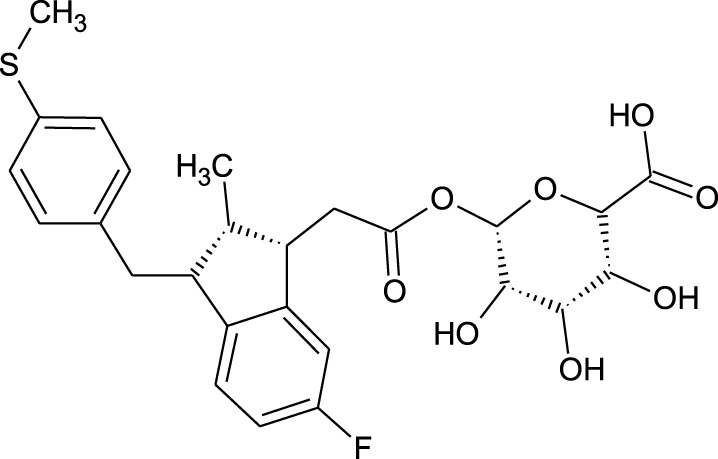
7	Clofazimine	−8.4	−7.6	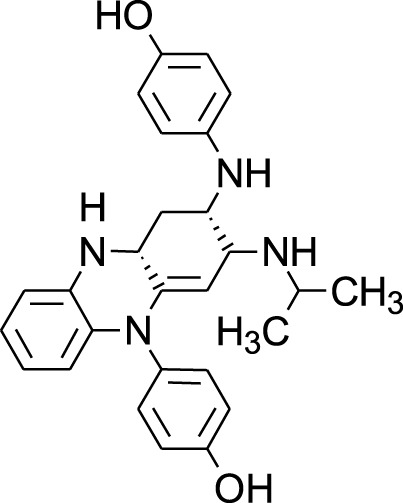
8	Permethrin	−6.8	−10.4	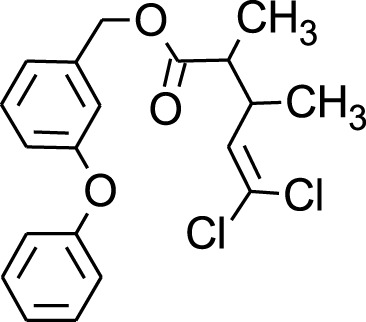
9	Deserpidine	−9.4	−10.7	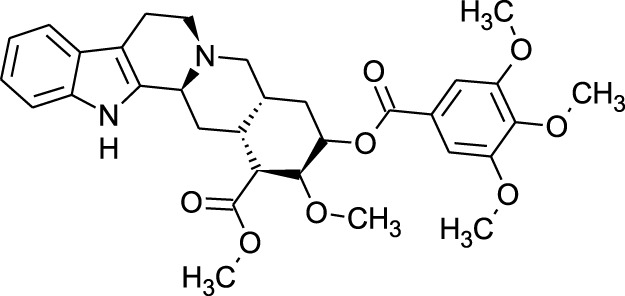
10	Convallatoxin	−8.4	−7.0	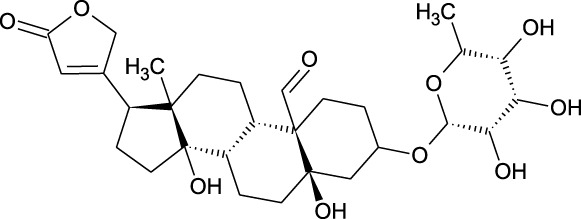
11	Perindoprilat	−7.7	−9.3	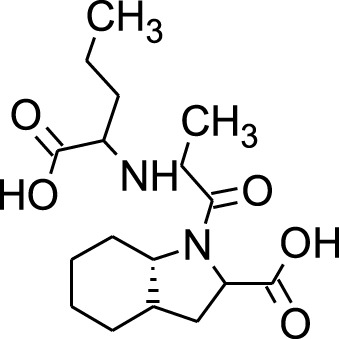
12	Hydroxycyclohexane -carboxylic acid	−5.5	−7.0	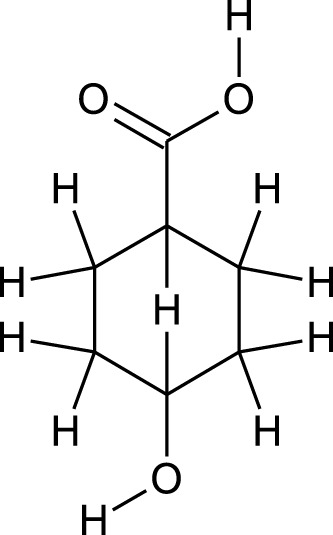
13	7,8- Dihydro- 14- hydroxynormorphine	−8.0	−9.9	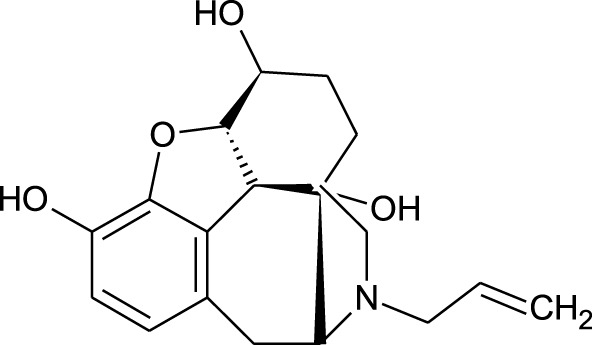
14	Asarylaldehyde	−5.3	−6.7	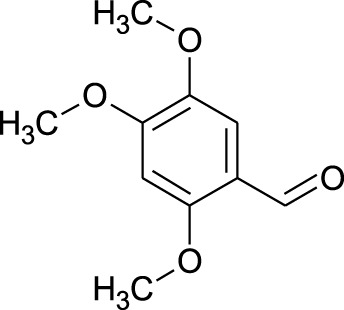
15	Nivalenol	−7.4	−7.2	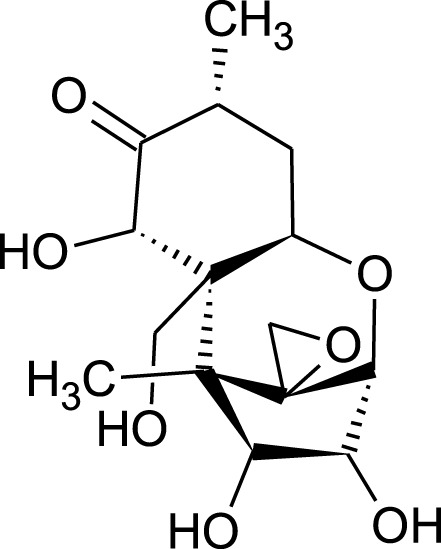
16	Ethoruximide M3 glucaronide	−7.7	−9.6	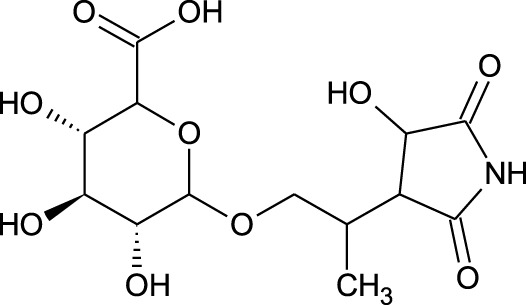
17	Epi- 4- hydroxyjarmonic acid	−7.7	−10.3	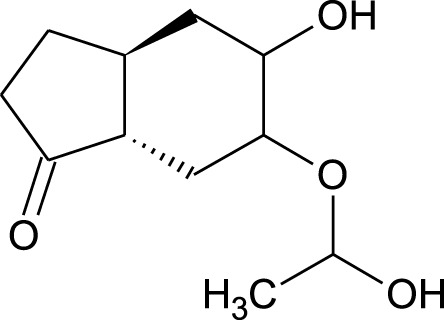
18	*N*- Acetylmuramic acid	−7.7	−8.7	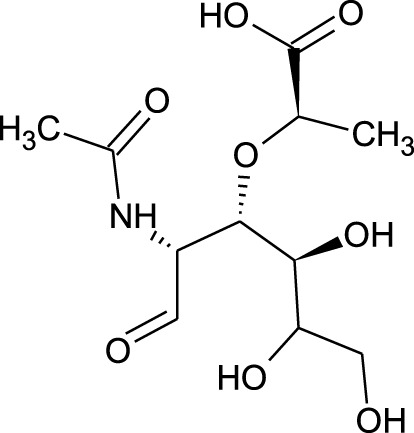
19	Lactone	−6.5	−8.0	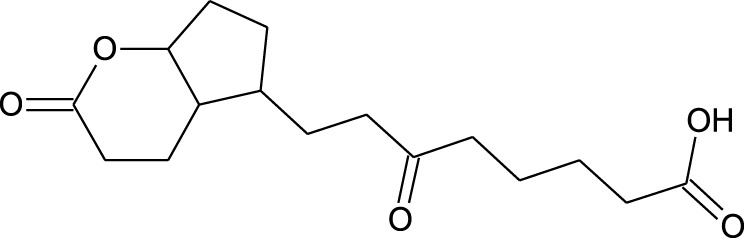
20	26,27- Diethyl- 1- alpha, 25- dihydroxyvitamin D3	−6.2	−7.1	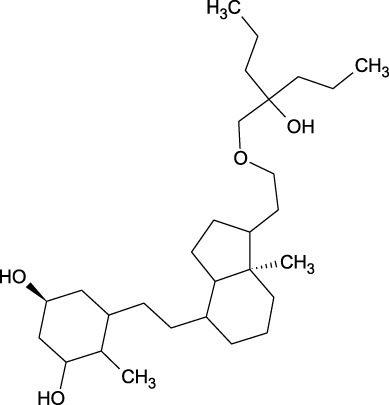
21	Carbenicillin	−6.9	−9.3	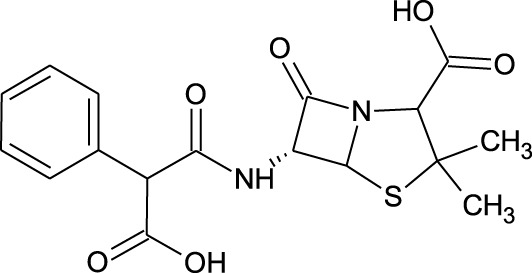
22	Rormarinic acid	−7.7	−9.3	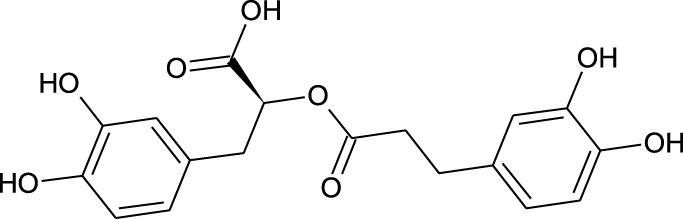
23	Methyl 8- octanoate	−7.3	−8.7	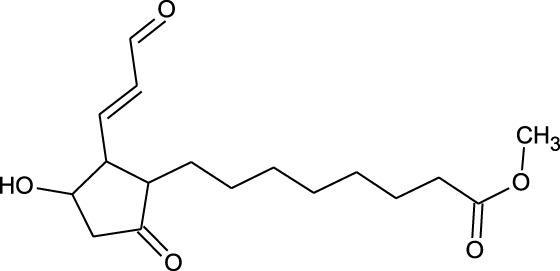
24	Idebenone	−6.8	−8.2	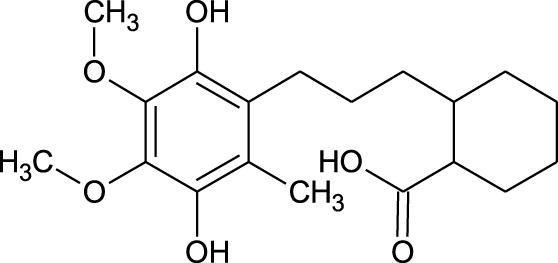
25	Gemfibrozil M1	−6.8	−9.0	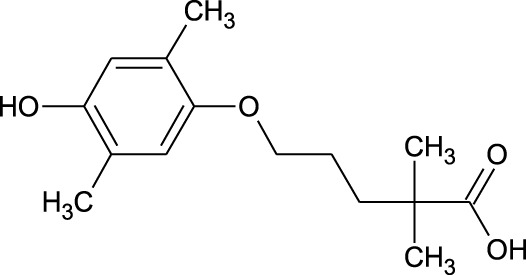

#### 2.5.3 Molecular docking studies

Evaluation of binding mode and its stability of ligand with BChE (4AQD) and AChE (4EY7) were performed using PyRx module of Autodock Vina. The two major steps of MD are an engine for sampling conformations and orientations and a scoring function that assigns a score to each projected position (Pradeep et al., 2021). The sampling procedure should seek the conformational space given by the free energy landscape, where energy is approximated by the scoring function in docking. The scoring function should be able to link the native bound-conformation to the energy hypersurface’s global minimum, as it will be used to separate putative valid binding modes and binders from non-binders in the pool of poses created by the sampling engine (Prasad et al., 2021).

The binding pockets of the respective proteins were set in the grid boxes. In case of BChE protein (PDB ID: 4AQD), the grid box measuring 39 Å × 39 Å × 39 Å was coordinated at x = 18.743,000, y = 27.587,000, and z = 11.264,000. Similarly, receptor AChE protein (PDB ID: 4EY7) was coordinated in a grid box of 34 Å × 34 Å × 34 Å dimensions at x = −29.345,000, y = 39.443,000, and z = 16.481,000.

It employs the Broyden-Fletcher-Goldfarb-Shanno (BGFS) method, which evaluates each ligand conformation’s scoring function for perturbation and local ligand optimization at the target location. In the MDS, ligands were treated as flexible while proteins were treated as stiff due to the high number of torsions generated during ligand formation. For ligand molecules, however, 10 degrees of freedom were allowed. Out of ten binding poses generated, the first one with zero root mean square deviation (RMSD) of atomic positions is declared fairly acceptable.

### 2.6 Molecular dynamics simulation studies

The GROMACS suite 2021 software was used to create a simulation of the ligand-enzyme combination. For molecular dynamics simulation (MDS), the ligand-enzyme complexes with the lowest binding energy were chosen. In the context of the CHARMM 27, the ligand parameters were examined utilizing the PRODRG online service (Prasad et al., 2021). Under periodic boundary circumstances, the ligand-enzyme complex was solvated utilising a simple point charge water box with 1.0 nm gap between the protein and the box faces. For complicated systems, Cl or Na + counter ions were used to neutralise the system. The steepest descent approach was used to reduce energy consumption for 1,000 steps. The systems were equilibrated for 100 ps at 300 K under constant number of particles, volume, and temperature circumstances, then for 100 ps under constant number of particles, pressure, and temperature conditions for the goal of flowing energy minimization (Srinivasa et al., 2022; Jain et al., 2021). The Linear Constraint Solver technique was used to limit all covalent connections with hydrogen atoms. The Particle Mesh Ewald approach was used to handle the electrostatic interactions. Finally, a 100 ns MD simulation was used to test the stability of the ligand-enzyme complexes (Prasad A. et al., 2020).

Using the GROMACS functions g_rmsd, g_rmsf, and g_hbond, the potential of each trajectory produced by MDS was investigated. The root mean square deviation (RMSD), root mean square fluctuation (RMSF), and the number of H-bonds formed between the ligand and the enzyme were calculated. The XM grace tool was used to create the graphs (Prasad et al., 2021).

### 2.7 Binding energy calculations

With the help of the Molecular Mechanics/Poisson-Boltzmann Surface Area (MM-PBSA) method, binding free energy calculations were performed on the results of the MDS run for all of the target proteins complexed with dehydronimodipine. To ascertain the degree of ligand interaction with protein, molecular dynamics simulations and thermodynamics are once again used. The binding free energy for each ligand-protein combination was calculated using the gmmpbsa software and MmPbStat.py script, which takes the GROMACS 2018.1 trajectories as input Prasad et al. (2020b). The binding free energy is determined by the g mmpbsa program using three different factors: molecular mechanical energy, polar and a polar solvation energies, and molecular mechanical energy. Using MDS, the calculation is completed. To compute ∆G with dt 1,000 frames, the latest 100 ns of trajectory were taken into consideration. It is assessed using polar and a polar solvation energies, as well as molecular mechanical energy. Below are Eqs 1, 2 that are used to determine the free binding energy (Avinash et al., 2021).

$$∆G_{Binding}=G_{Complex}-\left(G_{Protein}+G_{Ligand}\right)$$

$$∆G=∆E_{MM}+∆G_{Solvation}-T∆S=∆E_{\left(Bonded+nonbonded\right)}+∆G_{\left(Polar+nonpolar\right)}-T∆S$$

GBinding: binding free energy, GComplex: total free energy of the protein-ligand complex, GProtein and GLigand: total free energies of the isolated protein and ligand in solvent, respectively, ∆G: standard free energy, ∆EMM: average molecular mechanics potential energy in vacuum, ∆GSolvation: solvation energy, ∆E: total energy of bonded as well as non-bonded interactions, ∆S: change in entropy of the system upon ligand binding, T: temperature in Kelvin.

### 2.8 Density functional theory calculations

Frontier orbitals HOMO (Highest Occupied Molecular Orbital) and LUMO (Lowest Unoccupied Molecular Orbital) are intrinsically related to the chemical reactivity of the molecules, and can be calculated using the Kohn–Sham (KS) method, which calculates molecular energy, electronic density, and orbital energies (Young, 2001; Lewars, 2003; Cramer, 2004; Jensen, 2007). This methodology is convenient when thinking of quantitative qualities related with Conceptual DFT descriptors (Geerlings et al., 2003; Gazquez et al., 2007; Chattaraj et al., 2009; Geerlings et al., 2020). This methodology is convenient when thinking of quantitative qualities related with Conceptual DFT (Geerlings et al., 2020). The definitions for the global reactivity descriptors are (Chattaraj et al., 2009). Electronegativity as χ ≈ 1/2 (ϵ_L_ + ϵ_H_), Global Hardness as η ≈ (ϵ_L_ − ϵ_H_), Electrophilicity as ω ≈ (ϵ_L_ + ϵ_H_)^2^/4 (ϵ_L_ − ϵ_H_), Electrodonating Power as ω− ≈ (3ϵ_H_ + ϵ_L_)^2^/16η, Electroaccepting Power as ω+ ≈ (ϵ_H_ + 3ϵ_L_)^2^/16η and Net Electrophilicity as Δω = *ω* ^+^ − (−ω^−^) = *ω* ^+^ + *ω* ^−^. These global reactivity descriptors that arise from Conceptual DFT has been complemented by a Nucleophilicity Index N (Domingo et al., 2008; Jaramillo et al., 2008; Domingo and Sa´ez., 2009; Domingo and Perez., 2011; Domingo et al., 2016) that accounts for the HOMO energy level calculated with an arbitrarily shifted origin and tetracyanoethylene (TCE) as the reference.

Molecular Mechanics calculations using the comprehensiveMMFF94 force field were used in MarvinView 17.15, which can be obtained from ChemAxon [http://www.chemaxon.com], to determine the conformers of the molecules under consideration in the present study. Next, we optimized the geometry and determined the frequency using the Density Functional Tight Binding (DFTBA) method (Frisch et al., 2016). The stability of the optimized structures as the bare minimum in the energy landscape necessitated this last step of verifying the absence of imaginary frequencies. The MN12SX/Def2TZVP/H_2_O model chemistry (Weigend and Ahlrichs., 2005; Weigend, 2006; Tsuneda et al., 2010; Peverati and Truhlar., 2012; Tsuneda and Hirao., 2014; Kanchanakungwankul and Truhlar, 2021), based on its optimized molecular structure, is representative of the electronic properties and chemical reactivity descriptors of the molecules studied. Previous research using the Gaussian 16 program (Frisch et al., 2016) and the SMD solvation model (Marenich et al., 2009) has established that this method successfully validates the ‘Koopmans in DFT’ (KID) procedure (Frau and Glossman-Mitnik., 2018a; Flores-Holguín et al., 2019a; Frau et al., 2019; Flores-Holguín et al., 2019; Flores-Holguín et al., 2020a; Flores-Holguín et al., 2021). The radical anion and cation are considered to be in the doublet spin state, and the MN12SX screened-exchange density functional (Kar et al., 2013) is used alongside the Def2TZVP basis set (Weigend and Ahlrichs., 2005; Weigend, 2006) and a molecule with a charge of zero in this model chemistry.

## 3 Results and discussion

### 3.1 Characterization of AC-HAp-NPs

#### 3.1.1 SEM analysis

**FIGURE 2:**
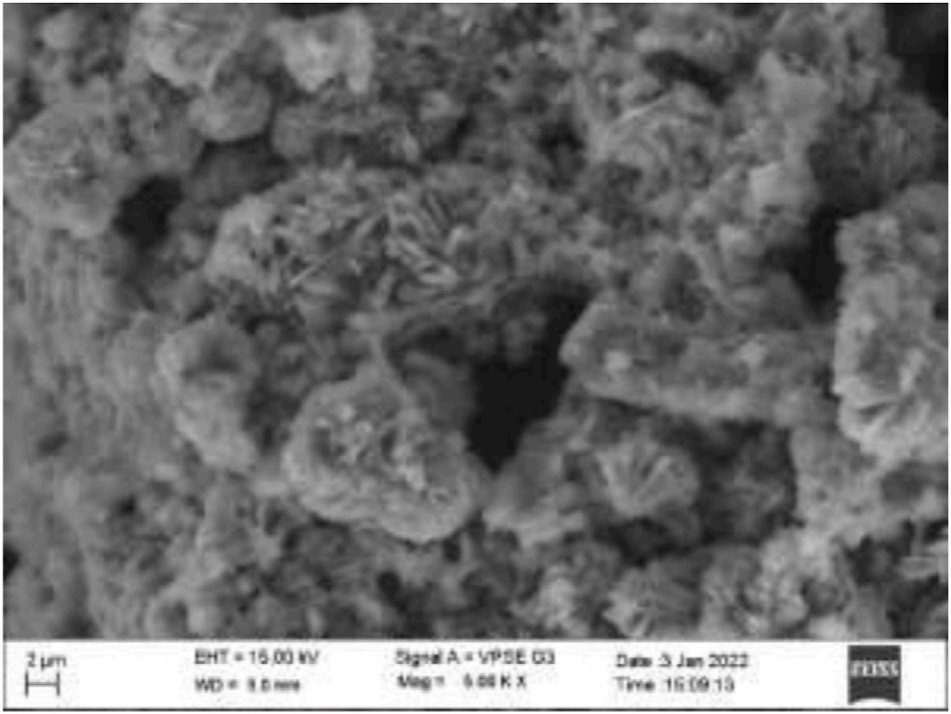
SEM image of *Areca Catechu* L. leaf extract derived hydroxy apatite nanoparticles showing nanoflake like morphology.

The surface morphology plays a crucial role in the application of AC-HAp-NPs. The SEM image of as-obtained AC-HAp-NPs revealed a nanoflake like morphology. It is noteworthy that the nanoflake hydroxy apatite nanoparticles have been recently used in bone cell attachment. The SEM results depicted that the average particles size ranged between 15 and 20 nm as showed in Figure 2 (Pradeep et al., 2021).

#### 3.1.2 EDX analysis

**FIGURE 3:**
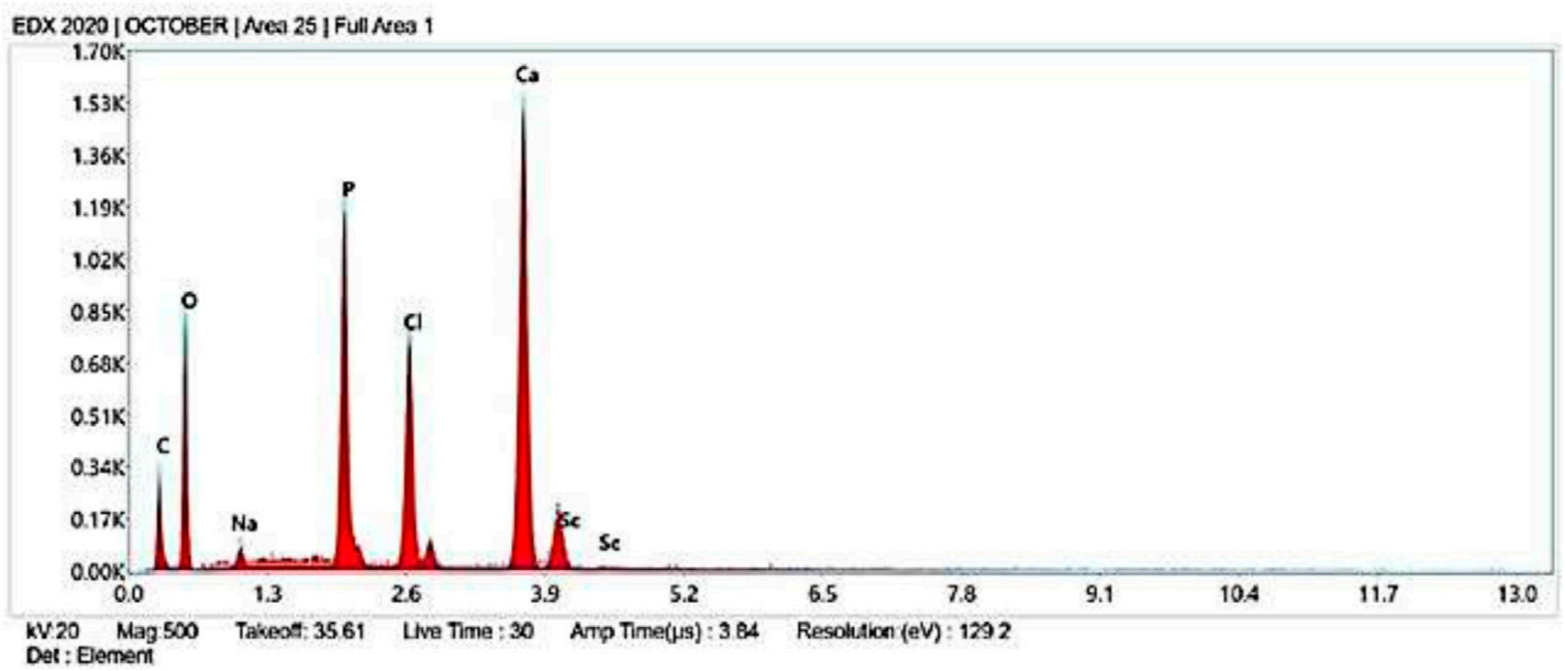
EDX images of *Areca Catechu* L. leaf extract derived hydroxy apatite nanoparticles.

The elemental composition of the AC-HAp NPs as they were obtained was confirmed by the EDAX analysis (Figure 3). The distinctive Ca, P, and O peaks seen in the EDAX spectra are plainly visible. The calcium to phosphate ratio, in particular, was found to be 1.69, which is close to the Ca/P ratio of human bone, according to (Pradeep et al., 2021). It is important to note that the atomic and weight percentages of the constituent particles. Further, the presence of different functional groups was confirmed by FT-IR analysis (Supplementary Figure S1).

#### 3.1.3 TEM analysis

**FIGURE 4:**
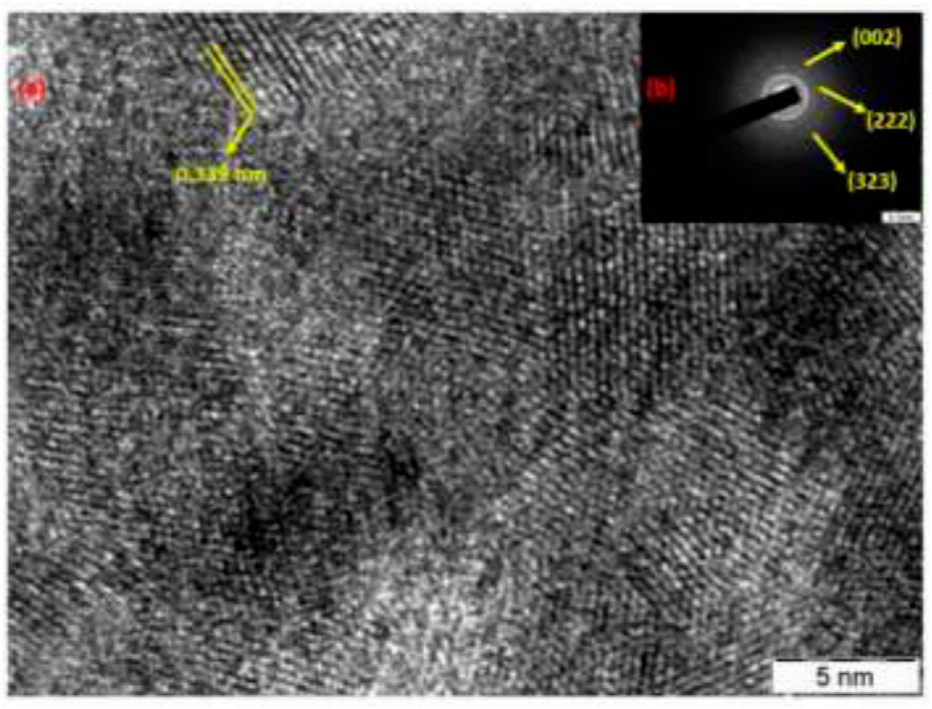
**(A)**HR-TEM and **(B)** SAED pattern of as-obtained AC-HAp-NPs (inset).

Figure 4 shows TEM images of as-obtained AC-HAp-NPs nanoflakes. TEM confirmed that the AC-HAp-NPs flakes are full of holes. The interplanar lattice spacings was found to be of 0.339 nm as shown in Figure 4A. Further, the SAED pattern (Figure 4B) revealed that this is in good agreement with the (002) interplanar distance (Pradeep et al., 2021).

#### 3.1.4 XRD analysis

**FIGURE 5:**
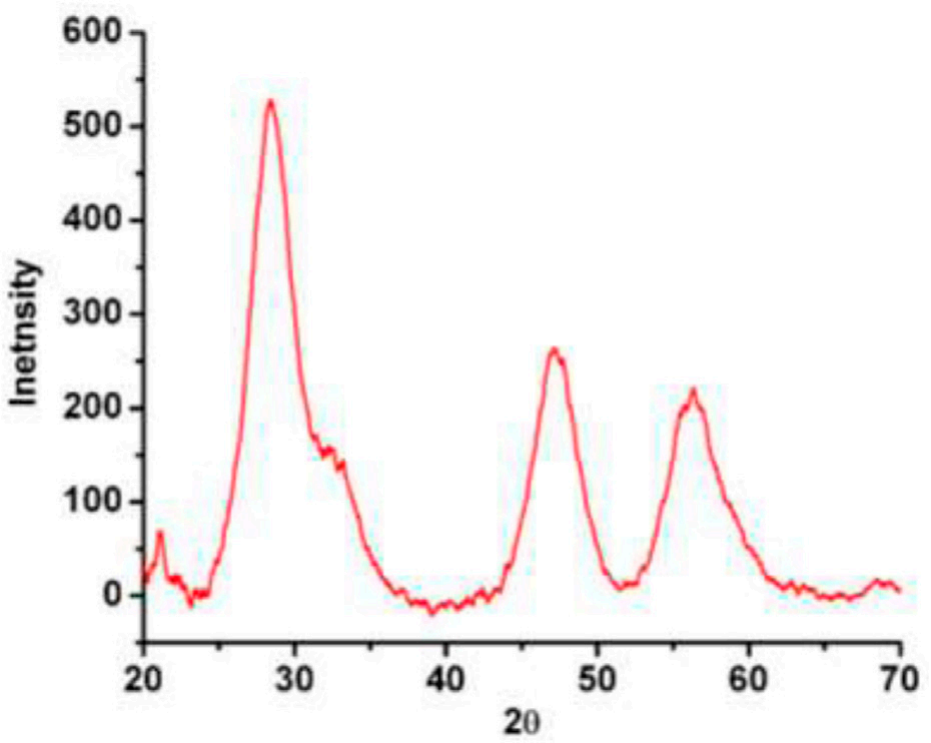
XRD pattern of as-obtained AC HAp-NPs.

XRD pattern of the resultant AC-HAp-NPs is shown in Figure 5

. The diffraction peaks at 2q = 28.09, 46.93 and 57.35°, corresponding to (002), 222) and 323) planes, respectively, which is in good agreement with the hexagonal system consisting primitive lattice. In addition, the average particle size was calculated using FWHM by Scherrer’ formula, D = kλ/βcosθ, which revealed that the as-obtained AC-HAp-NPs was about 35 mm (Pradeep et al., 2021).

### 3.2 AC-HAp NPs inhibits anti-AChE and anti-BChE activity

**FIGURE 6:**
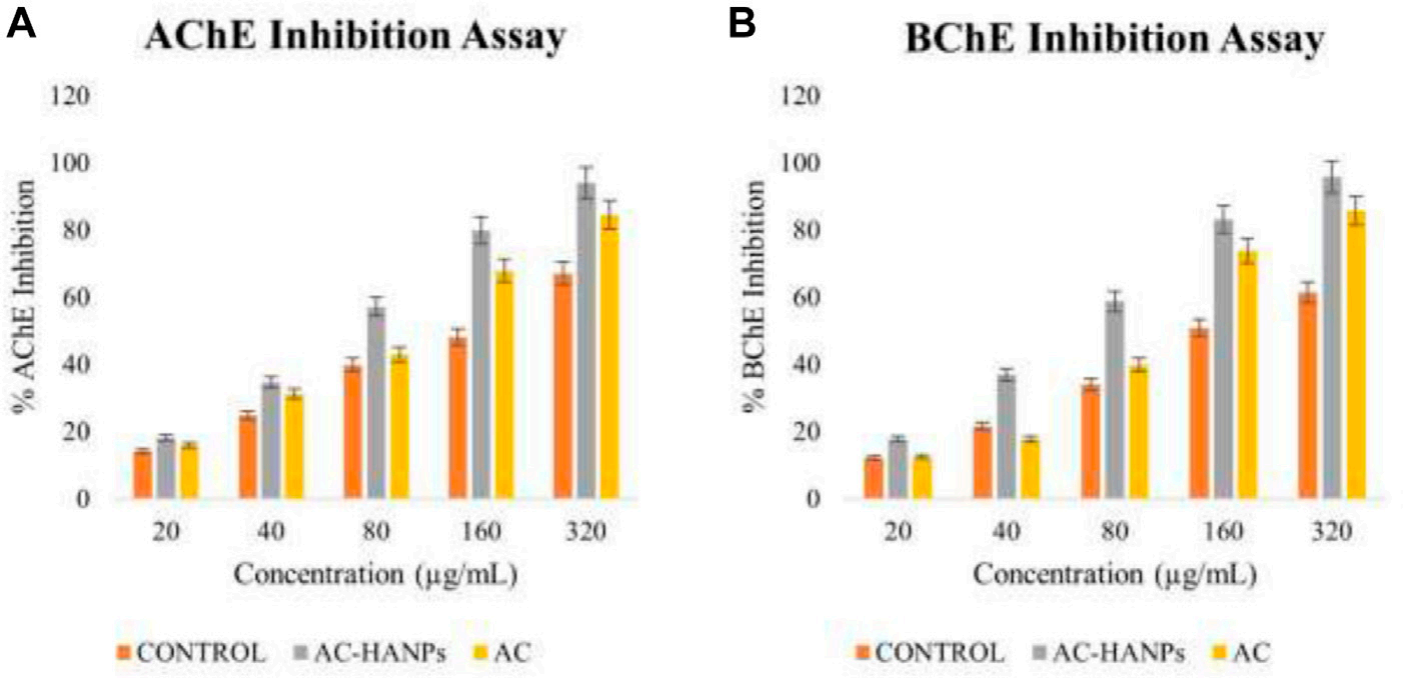
**(A)** Acetylcholinesterase (AChE) and **(B)** Butyrylcholinesterase (BChE) activity of AC-HANPs, AC extract and Tacrine as positive control in grey, yellow and orange respectively.

Inhibitors increase acetylcholine levels by inhibiting AChE and BChE of the cholinergic synapse, improving function and alleviating symptoms of neurological disorders such as AD. Plant-derived extracts are a key source of AChE and BChE inhibitors, in addition to alkaloid-derived compounds. AChE and BChE activity has been shown to be inhibited by HAp-NPs derived from the leaves of *Areca Catechu* L. As a result, AC-HAp-NPs from the AC leaf were discovered to inhibit both AChE and BChE. Surprisingly, AC-HAp-NPs had significantly higher anti-AChE and anti-BChE activity with an IC_50_ value of 79.92 (anti-AChE) and 82.5 μg/ml (anti-BChE) compared to positive control Tacrine (IC_50_ of 118.73 μg/ml) and leaf extract alone ((IC_50_ of 110.43 μg/ml) as represented in Figure 6.

### 3.3 *In silico* analysis

#### 3.3.1 Protein binding site prediction

**FIGURE 7:**
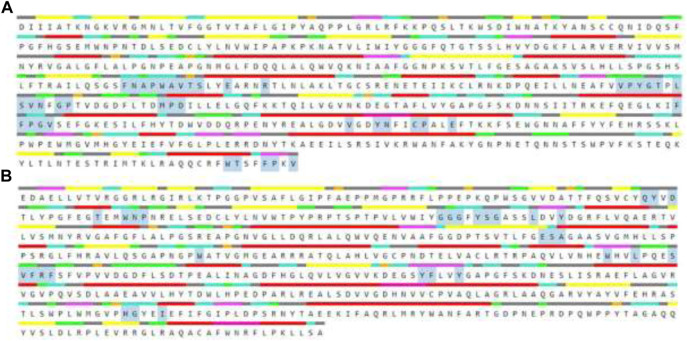
The amino acid residues highlighted in grey specifies the binding pocket of the selected receptors **(A)** BChE (4AQD) and **(B)** AChE (4EY7).

Protein binding sites are defined as residues in a protein that interact with a ligand. The CASTp 3.0 server, which stands for Computed Atlas of Surface Topography of Proteins (http://sts.bioe.uic.edu/castp/index.html?4jii), was used to anticipate this binding site (Figure 7). CASTp detects and measures surface pockets and internal cavities. The server identifies the amino acids that are important for binding interactions, and the modelled protein is utilized to predict ligand binding sites (Mariwamy et al., 2022).

#### 3.3.2 Molecular docking studies

**FIGURE 8:**
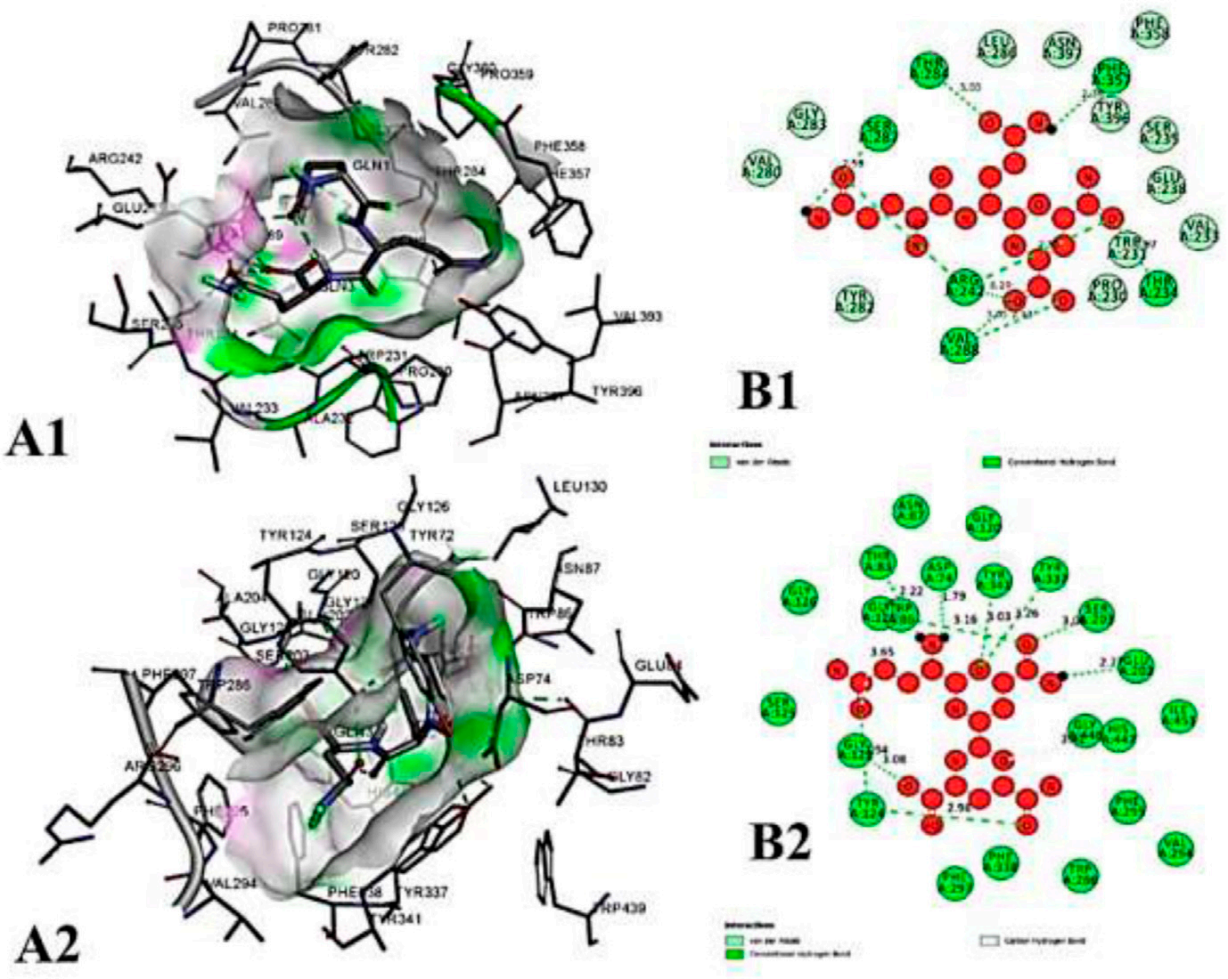
The molecular docking interactions of ligand dehydronimodipine with **(A)** 4AQD and **(B)** 4EY7 receptors in 2D and 3D representation.

**FIGURE 9:**
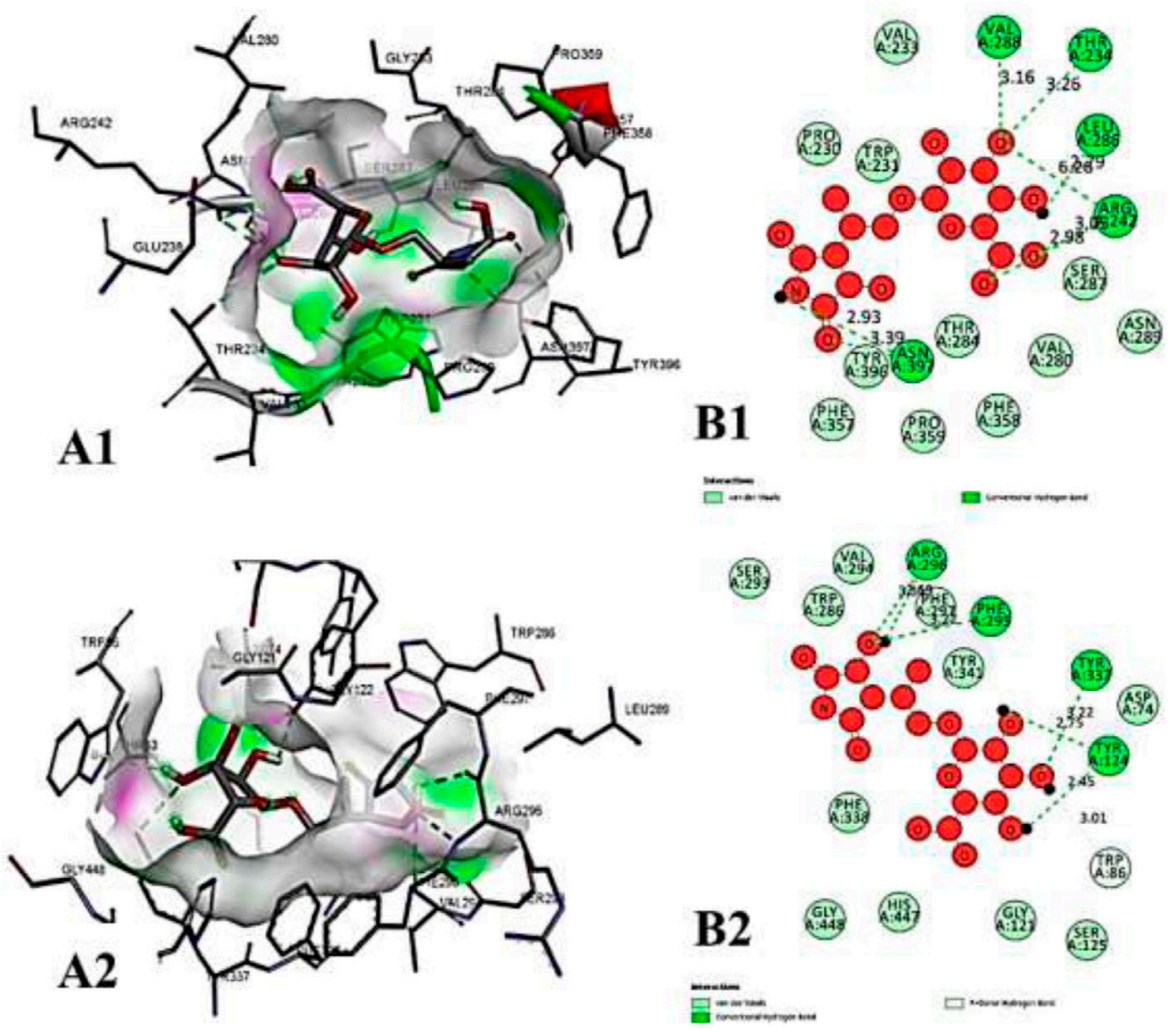
The molecular docking interactions of ligand *N*- acetylmuramic acid with **(A)** 4AQD and **(B)** 4EY7 receptors in 2D and 3D representation.

**FIGURE 10:**
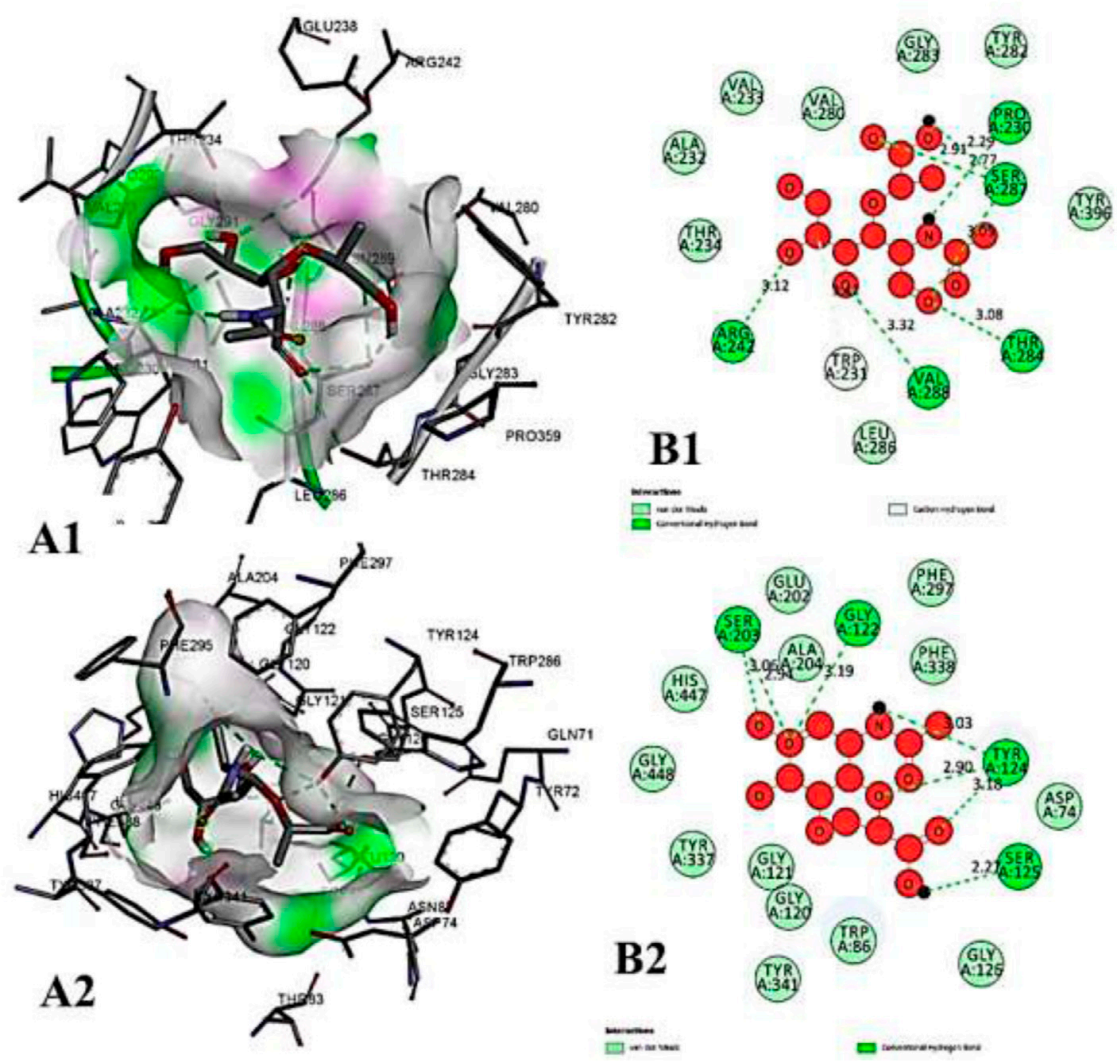
The molecular docking interactions of ligand 26,27- diethyl- 1- alpha, 25- dihydroxyvitamin D3 with **(A)** 4AQD and **(B)** 4EY7 receptors in 2D and 3D representation.

**FIGURE 11:**
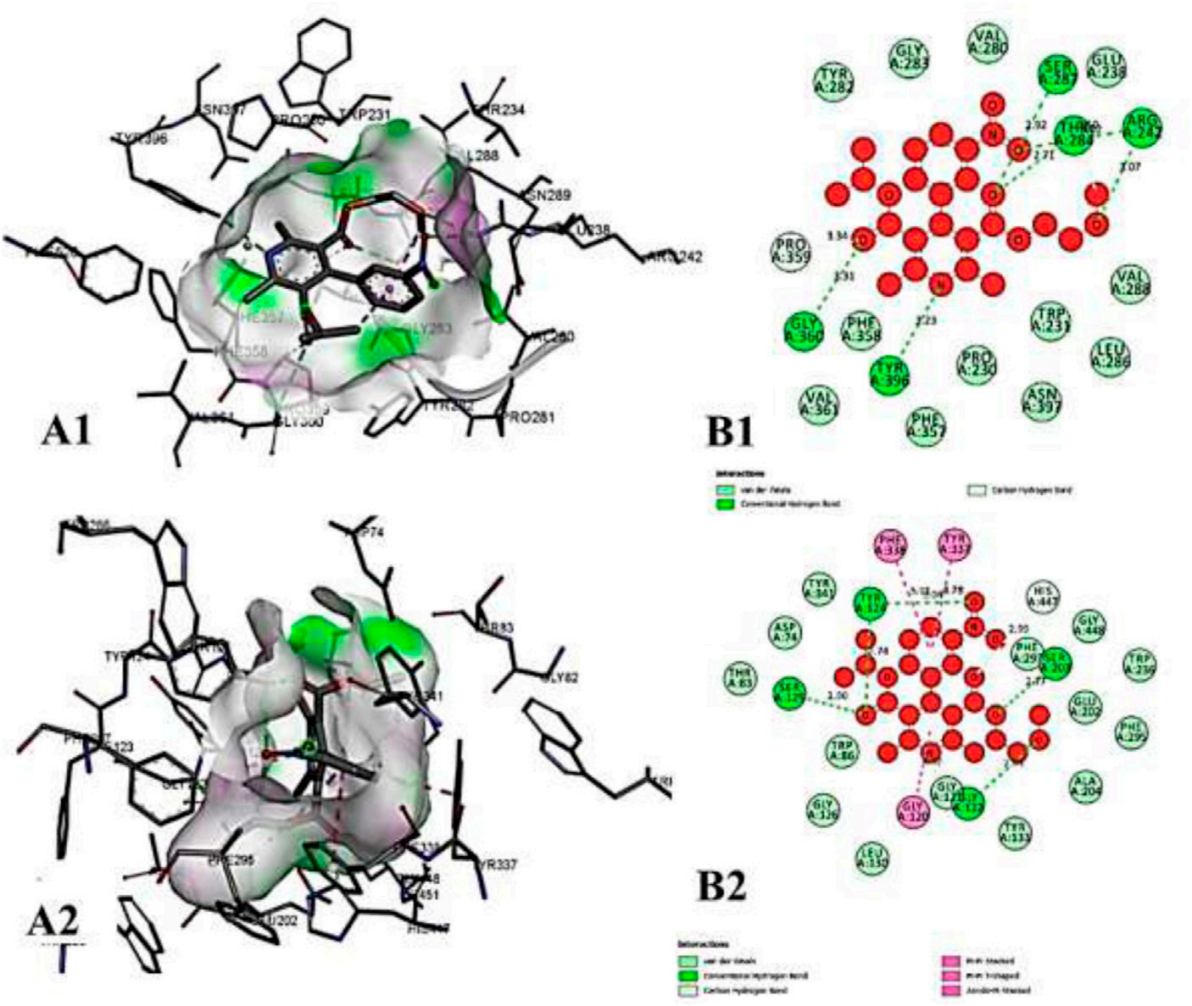
The molecular docking interactions of ligand *N*-acetylcilartatin with **(A)** 4AQD and **(B)** 4EY7 receptors in 2D and 3D representation.

**FIGURE 12:**
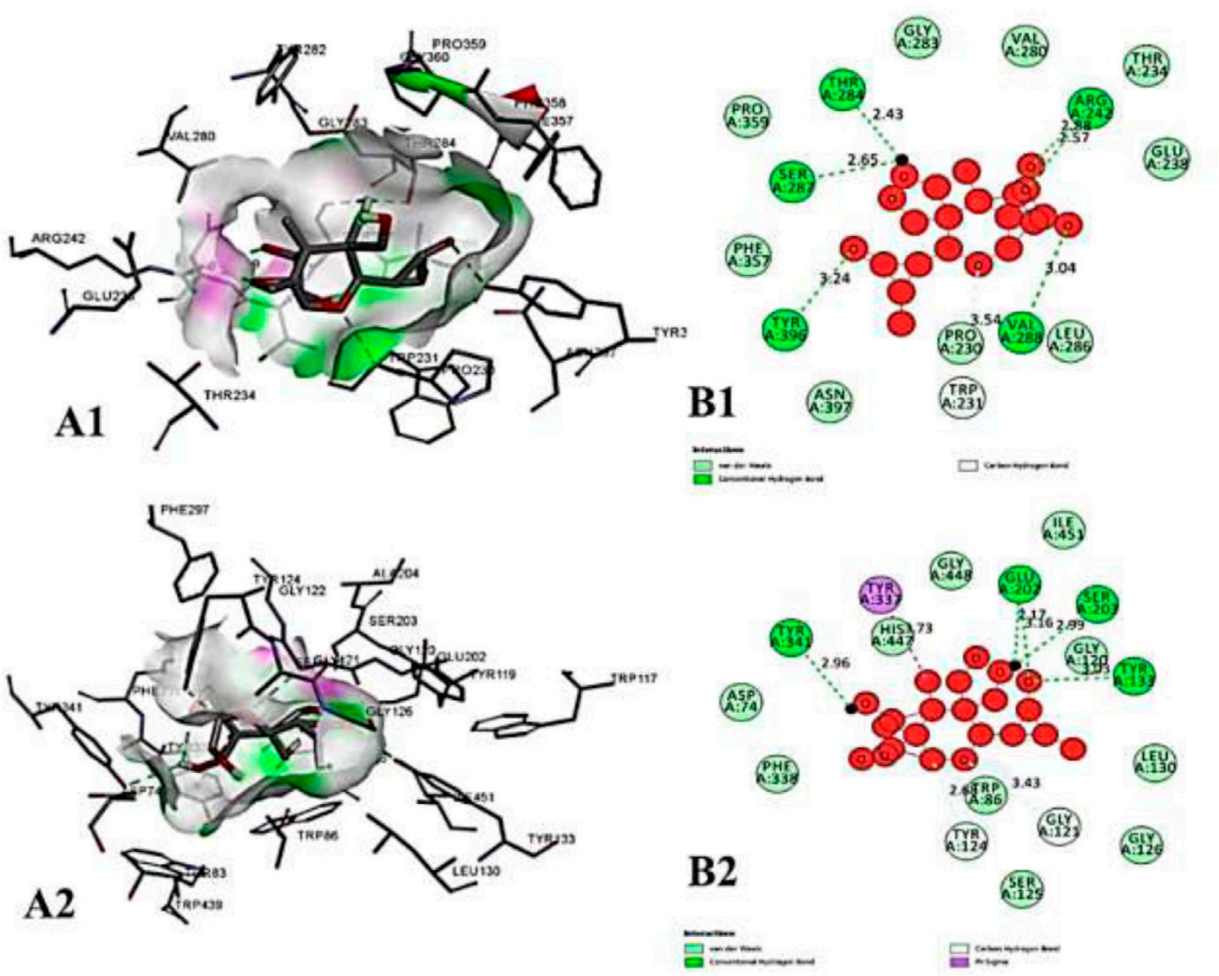
The molecular docking interactions of ligand nivalenol with **(A)** 4AQD and **(B)** 4EY7 receptors in 2D and 3D representation.

We compiled a list of ligand binding conformations based on binding energy from docking studies. The binding conformation of the compounds to the chosen proteins was identified, and among the possible conformations, the one with the lowest binding energy was produced (Uppar et al., 2021). Among the 25 ligands the molecular interactions of top five compounds with least binding energy and highest binding affinity of dehydronimodipine, *N*- acetylmuramic acid, 26,27- diethyl- 1- alpha, 25- dihydroxyvitamin D3, *N*-acetylcilartatin and nivalenol figures have been represented from Figures 8–12 and their respective bonded and non-bonded interaction details are given in Tables 2, 3. In comparison to higher energy values, lower energy scores indicate better protein-ligand binding affinity. Among all the 25 compounds the ligand dehydronimodipine has the least binding affinity of −11.7 kcal/mol with and AChE (4EY7), binding energy value with BChE (4AQD) was −10.1 kcal/mol.

**TABLE 2:** Molecular docking binding energy and interactions of five ligands against 4AQD.

Ligand no	Molecular name	Bonded interactions	Non-bonded interactions
4	Dehydronimodipine	9 interactions	PRO-230, TRP-231, VAL-233, SER-235, GLU-238, VAL-280, TYR-282, GLY-283, LEU-286, PHE-358, TYR-396, ASN-397
		THR-234, ARG-242, THR-284, SER-287, VAL-288, PHE-357	
18	*N*- Acetylmuramic acid	8 interactions	PRO-230, TRP-231, VAL-233, VAL-280, THR-284, SER-287, ASN-289, PHE-357, PHE-358, PRO-359, TYR-396
		THR-234, ARG-242, LEU-286, VAL-288, ASN-397	
20	26,27- Diethyl- 1- alpha, 25- dihydroxyvitamin D3	7 interactions	TRP-231, ALA-232, VAL-233, THR-234, VAL-280, TYR-282, GLY-283, LEU-286, TYR-396
		PRO-230, ARG-242, THR-284, SER-287, VAL-288	
5	*N*-Acetylcilartatin	8 interactions	PRO-230, TRP-231, GLU-238, VAL-280, TYR-282, GLY-28a3, LEU-286, VAL-288, PHE-357, PHE-358, PRO-359, VAL-361, ASN-397
		ARG-242, THR-284, SER-287, GLY-360, TYR-396	
15	Nivalenol	7 interactions	THR-234, GLU-238, VAL-280, GLY-283, LEU-286, PHE-357, PRO-359, ASN-397
		PRO-230, TRP-231, ARG-242, THR-284, SER-287, VAL-288, TYR-396	

**TABLE 3:** Molecular docking binding energy and interactions of five ligands against 4EY7.

Ligand no	Molecular name	Bonded interactions	Non-bonded interactions
4	Dehydronimodipine	12 interactions	ASN-87, GLY-120, SER-125, GLY-126, TRP-286, PHE-295, PHE-297, VAL-294, PHE-295, HIS-447, ILE-451
		ASP-74, THR-83, TRP-86, GLY-121, GLY-122, TYR-124, GLU-202, SER-203, TYR-337, TYR-341	
18	*N*- Acetylmuramic acid	7 interactions	ASP-74, GLY-121, SER-125, TRP-286, SER-293, VAL-294, PHE-297, PHE-338, TYR-341, HIS-447, GLY-448
		TRP-86, TYR-124, PHE-295, ARG-296, TYR-337	
20	26,27- Diethyl- 1- alpha, 25- dihydroxyvitamin D3	7 interactions	ASP-74, TRP-86, GLY-120, GLY-121, GLY-126, GLU-202, ALA-204, PHE-297, TYR-337, PHE-338, TYR-341, HIS-447, TYR-448
		GLY-122, TYR-124, SER-125, SER-203	
5	*N*-Acetylcilartatin	6 interactions	ASP-74, THR-83, TRP-86, GLY-121, GLY-126, LEU-130, TYR-133, GLU-202, ALA-204, TRP-236, PHE-295, PHE-297, TYR-337, PHE-338, TYR-341, GLY-448
		GLY-122, TYR-124, SER-125, SER-203, HIS-447	
15	Nivalenol	7 interactions	ASP-74, TRP-86, SER-125, GLY-120, GLY-126, LEU-130, TYR-337, PHE-338, HIS-447, GLY-448, ILE-451
		GLU-202, SER-203, GLY-121, TYR-124, TYR-133, TYR-341	

##### 3.3.2.1 Hydrogen bond interactions

The ligand dehydronimodipine propensity for binding to proteins was studied in depth. The residues ASP-74 (1.79Å), THR-83 (2.22Å), TRP-86 (3.16Å, 2.22Å), GLY-121 (3.65Å), GLY-122 (3.08Å), TYR-124 (2.98Å, 2.94Å), GLU-202 (2.27Å), SER-203 (3.00Å), TYR-337 (3.26Å) and TYR-341 (3.03Å) are in H-bond contact with the ligand and share twelve hydrogen bonds, according to an analysis of the ligand binding mode into the catalytic site of 4EY7. The residues THR-234 (2.87Å), ARG-242 (2.85Å, 3.03Å), THR-284 (3.00Å), SER-287 (2.58Å), VAL-288 (2.93Å, 3.05Å) and PHE-357 (2.38Å) are implicated in the H-bond interaction, and they provided eight hydrogen bonds, according to the binding mechanism of ligand with 4AQD. The interaction of dehydronimodipine with 4AQD and 4EY7 reveals that both polar and aromatic amino acids play a key role in the ligand binding relationship.

##### 3.3.2.2 Hydrophobic interactions

The ligand-enzyme interaction is significantly influenced by hydrophobic interactions. Using the visualization tool Discovery Studio, the residues of BChE 4AQD and AChE (4EY7) involved in the hydrophobic interaction with ligand were examined. In dehydronimodipine-4EY7 complex analysis, ASN-87, GLY-120, SER-125, GLY-126, TRP-286, PHE-295, PHE-297, VAL-294, PHE-295, HIS-447 and ILE-451 were formed hydrophobic bonds with ligand. From the dehydronimodipine-4AQD complex analysis, it is found that the residues PRO-230, TRP-231, VAL-233, SER-235, GLU-238, VAL-280, TYR-282, GLY-283, LEU-286, PHE-358, TYR-396 and ASN-397 are participating in hydrophobic interactions with ligand. In contrast to the other AChE (4EY7), more residues in the two complexes contributed to the hydrophobic contact with BChE 4AQD.

In contemporary structure-based medication design, *in silico* MD has proven to be an effective approach. AC demonstrated effective inhibition in this anticholinesterase screening testing. Here, the docking study was undertaken to discover the responsible molecules from AC on anticholinesterase potential. However, evidence for our claimed biological consequence may be found in the binding affinities and binding poses of particular chemicals with the AChE and BChE. All of the ligands shown strong binding affinities, and some of them interacted with the protein’s catalytic site. The drugs with the highest binding affinities to AChE and BChE were dehydronimodipine, *N*-acetylmuramic Acid, 26,27-diethyl-1-alpha, 25-dihydroxy, *N*-acetylcilartatin, and nivalenol.

#### 3.3.3 Molecular dynamics simulation studies

The protein-ligand complex’s dynamic behaviour with respect to time in a solvated environment was investigated using MDS in addition to docking. Therefore, the stability of these complexes under simulated conditions was mapped using the reference ligand and the best active developed ligand. The simulation study provides analysis of the secondary structure pattern between the protein and their complexes as well as the root mean square deviation (RMSD), radius of gyration (Rg), solvent accessible surface area (SASA), ligand RMSD, and number of ligand hydrogen bonds maintained throughout the simulation time.

The RMSD of protein-ligand complex depicts the extent of stability of the same throughout the simulation. The radius of gyration (Rg) considers the varied masses calculated to root mean square distances considering the central axis of rotation. It considers the capability, shape and folding during each time step of the whole trajectory throughout the simulation. The protein structural areas that deviate most/least from the mean are the focus of the RMSF. Additionally, by measuring root mean square distances from the rotational axis. Solvent accessible surface area (SASA) measures the area around the hydrophobic core formed between protein-ligand complexes. Further, ligand RMSD depicts the stability of ligand during the simulation process. In addition, H-bonds appear during the whole simulation period of the molecular docking study that is being examined. During the analysis, all intermolecular H-bonds between the ligands and the specific protein were taken into account and shown appropriately. Independently, 4 simulations were performed at 100 ns time with the native protein alone and in complex with the dehydronimodipine. The primary goal of performing MD simulation is to understand binding affinities at a time-bound stability among ligands to proteins bound complexes.

**FIGURE 13:**
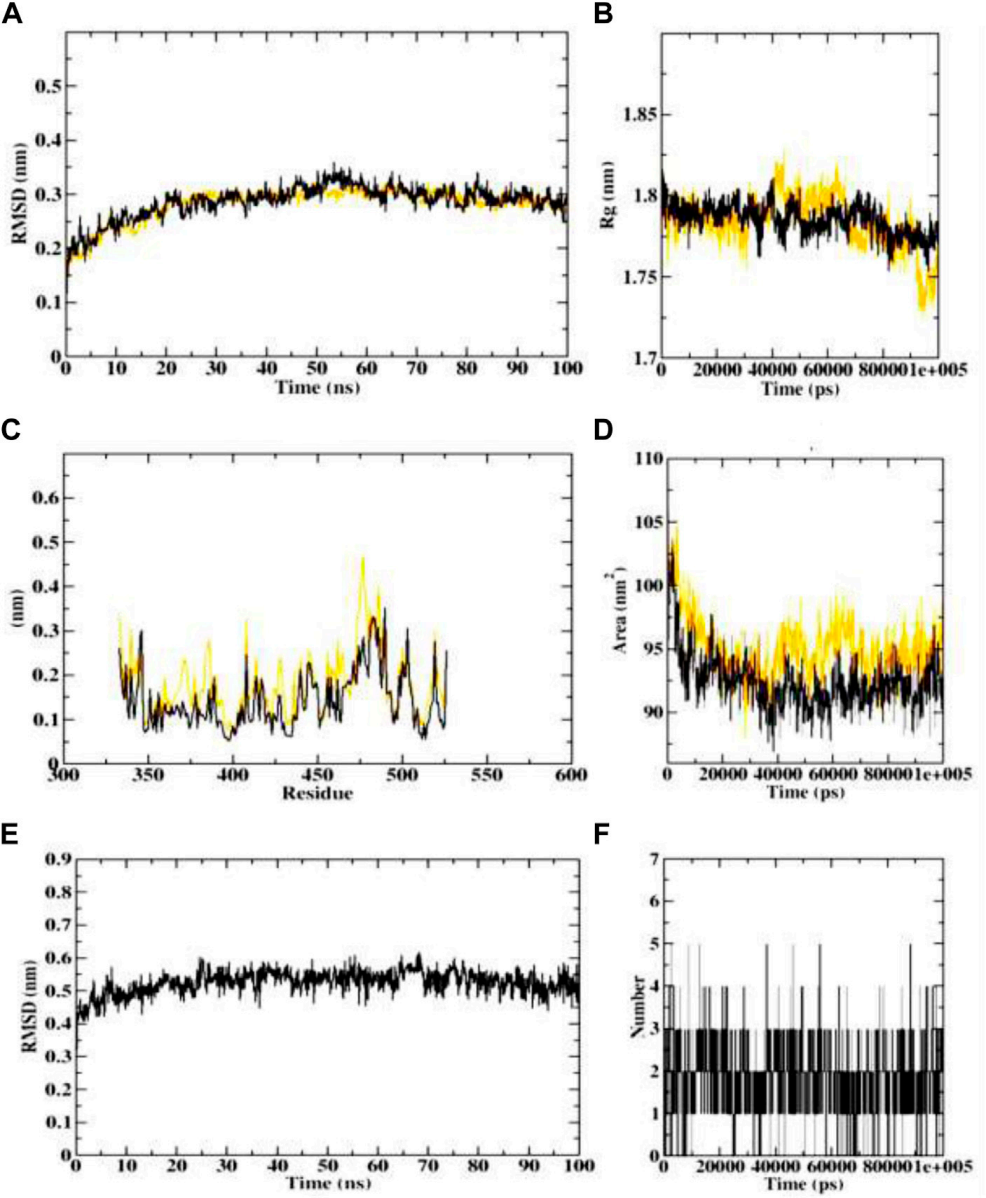
Visualization of MD trajectories of dehydronimodipine complexed with 4EY7 Run for 100 ns **(A)** protein-ligand complex RMSD, **(B)** protein-ligand complex Rg, **(C)** protein-ligand complex RMSF, **(D)** protein-ligand complex SASA, **(E)** ligand RMSD, **(F)** ligand hydrogen bonds. (red: protein backbone atoms, black: protein-ligand complex).

In case of AChE (4EY7), the protein-ligand complex RMSD plots depict that both protein backbone atoms and protein-ligand complexes were found to be completely stable after 20 ns. both the plots were concurrent throughout the simulation period, and equilibrated at 0.3 nm. The Rg plots of both the protein and protein-ligand complex also equilibrated within 1.75–1.8 nm, indicating the stability of the protein-ligand complex. Both the SASA plots of protein and protein-ligand complex were predicted with the value of 90–95.5 nm^2^, indicating the consistency in complex formation. Further, ligand RMSD also depicted the stability of the dehydronimodipine, by getting stable after 20 ns and equilibrating at 0.5 nm. Furthermore, maximum number of ligand hydrogen bonds were predicted to be 12 as shown in Figure 13.

**FIGURE 14:**
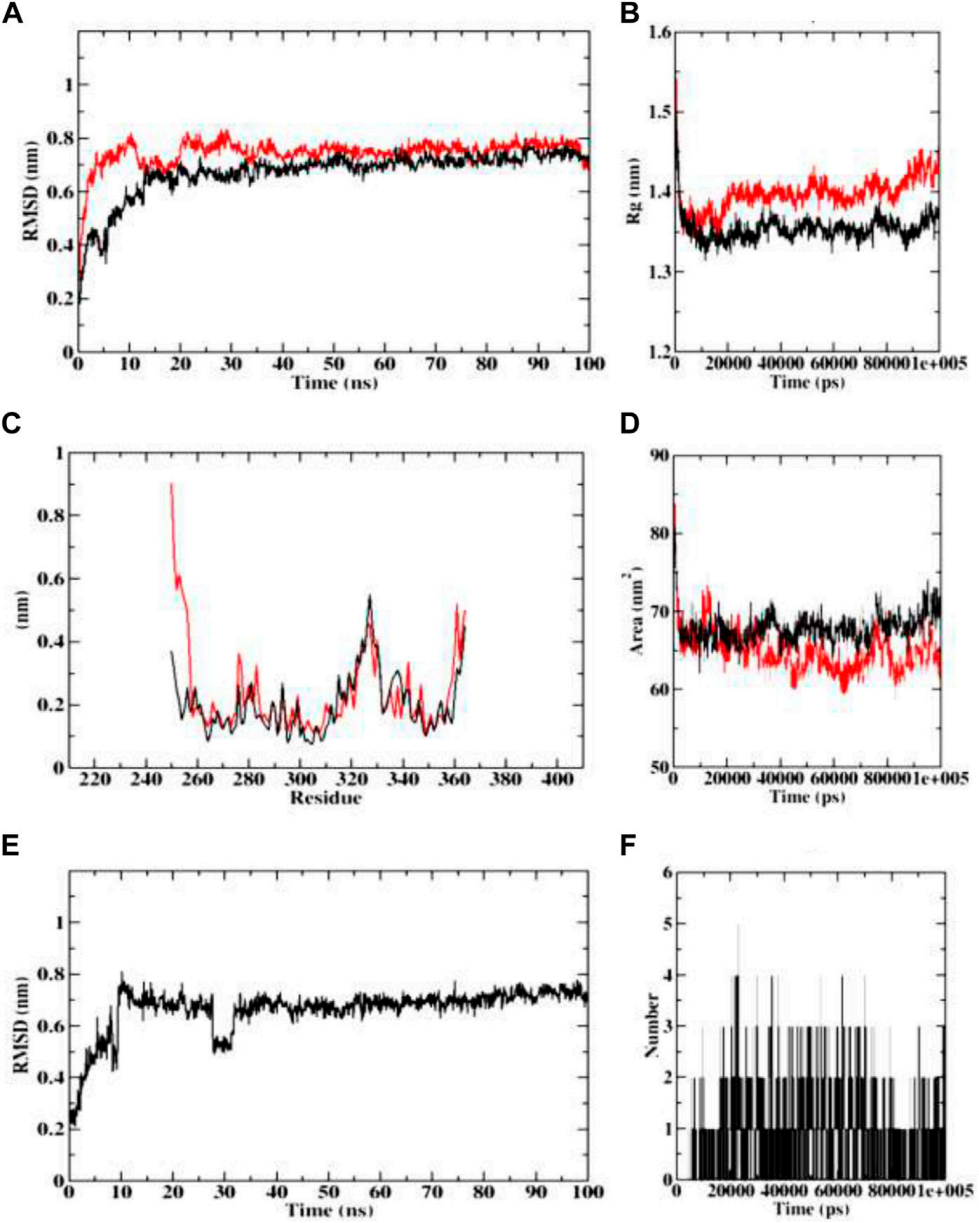
Visualization of MD trajectories of dehydronimodipine complexed with 4AQD Run for 100 ns **(A)** protein-ligand complex RMSD, **(B)** protein-ligand complex Rg, **(C)** protein-ligand complex RMSF, **(D)** protein-ligand complex SASA, **(E)** ligand RMSD, **(F)** ligand hydrogen bonds. (red: protein backbone atoms, black: protein-ligand complex).

In course of dynamics simulation of BChE (4AQD), the RMSD plots of both the protein backbone atoms and protein-ligand complexes were found to become stable after initial fluctuations at 20 ns The concurrent plots of both the molecular entities are visible without major fluctuations throughout the simulation process, and predict the stable interaction of dehydronimodipine with the BChE protein. Finally, both the plots equilibrate at 0.7 nm, representing structural flexibility of proteins being reserved when in complex with ligands than in the free form. The Rg plots also indicate the stability and reports no abnormality in protein-ligand complex formation. Both the protein backbone and protein-ligand complexes were predicted with the Rg value ranging between 1.35 and 1.45 nm. The RMSF plots were predicted with fluctuations only at the terminal ends and loop regions, indicating the stable interactions between protein and ligand. The similar pattern was observed in case of SASA plots, showing the range of 60–75 nm 2 area, which depicts the consistency of the protein-ligand complex formation throughout the simulation. Further, ligand RMSD plot indicated that the ligand became stable after the initial fluctuation at 10 ns However, the ligand continues to achieve stability despite a setback in stability at 30 ns, reaching equilibration at 0.7 nm. Furthermore, dehydronimodipine was found to form a maximum of 9 hydrogen bonds during the simulation process as shown in Figure 14.

#### 3.3.4 Binding free energy calculations

Various energy metrics such as Van der Waal’s, electrostatic, polar solvation, SASA, and binding energies are utilized to measure the extent of ligand-target protein binding interactions during molecular dynamics simulations. In this study, the Van der Waal energy was primarily used to construct the protein-ligand combination. Electrostatic energy, SASA energy, and binding energy came next. Polar solvation energy was predicted with no contribution for the protein-ligand complex formation, as the values appeared in positive. The AChE (4EY7) and BChE (4AQD) complexed with dehydronimodipine was predicted with the highest binding affinity and hence was considered for binding energy calculation studies. In addition, the protein-ligand complex standard deviations were calculated. A lower standard deviation means the data values are closer to the mean (or expected value), whereas a high standard deviation means the data values are spread out over a wider range. However, in the complex there was no high standard deviations. This indicates that dehydronimodipine binds to the protein with high binding affinity and stable interaction. The binding free energy calculations of protein-ligand complex have been given in Table 4.

**TABLE 4:** Binding free energy calculations of 4EY7 and 4AQD target protein complexed with complexed with dehydronimodipine.

Categories	4EY7- dehydronimodipine complex		4AQD- dehydronimodipine complex	
	Values (kj/mol)	Standard deviation (kj/mol)	Values (kj/mol)	Standard deviation (kj/mol)
Van der Waal’s energy	−189.746	+/−159.137	−152.635	+/−152.976
Electrostatic energy	−45.672	+/−39.198	−41.095	+/−34.088
Polar solvation energy	78.164	+/−68.852	84.734	+/−67.923
SASA energy	−23.120	+/−15.227	−24.459	+/−14.872
Binding energy	−145.440	+/−129.163	−112.873	+/−11.566

### 3.4 Conceptual DFT report

The quality of a density functional can be assessed by contrasting its predictions with either experimental data or findings from more advanced calculations. However, this comparison is sometimes computationally problematic because of a lack of experimental results for the molecular systems under consideration or because the molecules themselves are too big. A methodology called KID (Koopmans in DFT) has been developed by our research group (Frau and Glossman-Mitnik., 2018b; 2018c; 2018d; 2018e; 2018f; Flores-Holguín et al., 2019a; 2019b; 2019c; Flores-Holguín et al., 2020a; 2020b; 2020c; Flores-Holguín et al., 2021), for the validation of a given density functional in terms of its internal coherence. Based on prior work showing that the MN12SX/Def2TZVP/H_2_O model chemistry is superior to other density functionals (Flores-Holguín et al., 2022) like LC-ωHPBE, CAM-B3LYP, and ωB97XD when it comes to fulfilling and Ionization theorems, especially when studying large molecules, we have chosen this model chemistry for our computational determinations of the CDFT reactivity descriptors. In spite of this, we believe it is important to offer further support for the application of this model chemistry by estimating the KID parameters for the molecules under investigation and confirming that their values are close to zero. Table 5 displays the outcomes of these calculations.

**TABLE 5:** Frontier orbital energies, HOMO-LUMO gap and the KID descriptors for the studied molecules (all in eV).

Molecule	HOMO	LUMO	SOMO	H-L gap	J_*I*_	J_*A*_	^J^ *HL*	∆SL
MOL1	−7.27	−3.71	−3.40	3.56	0.280	0.588	0.652	0.307
MOL2	−6.91	−1.18	−1.05	5.73	0.216	0.126	0.250	0.138
MOL3	−6.14	−1.90	−1.67	4.25	0.714	0.373	0.805	0.225
MOL4	−7.08	−2.20	−1.89	4.88	0.119	0.414	0.431	0.309
MOL5	−5.68	−1.23	−1.06	4.45	0.275	0.324	0.425	0.173

Where MOL1 is dehydronimodipime, MOL2 is *N*- acetylmuramic acid, MOL3 is *N*-acetylcilastatin, MOL4 is nivalenol, and MOL5 is 26,27-diethyl- one- alpha, 25-dihydroxyvitamin D3.

The results for the KID descriptors present in Table 5 indicate that the MN12SX/Def2TZVP/H_2_O model chemistry describes adequately the behavior of the molecular system in relation to the verification of Ionization Energy theorem within the Generalized Kohn–Sham (GKS) model.

The results for the determination of the conceptual DFT reactivity descriptors for the studied molecules are displayed in Table 6.

**TABLE 6:** Global reactivity descriptors for the studied molecules (all in eV), but Softness S, in eV^−1^.

Molecule	χ	η	ω	S	N	ω^−^	ω^+^	∆ω^±^
MOL1	5.49	3.56	4.23	0.28	1.52	11.43	5.94	17.38
MOL2	4.05	5.73	1.43	0.17	1.88	5.24	1.19	6.45
MOL3	4.02	4.25	1.90	0.24	2.65	6.08	2.06	8.14
MOL4	4.64	4.88	2.21	0.20	1.71	7.04	2.40	9.43
MOL5	3.46	4.45	1.34	0.22	3.11	4.70	1.24	5.93

The results for the global reactivity descriptors derived from CDFT for the five studied molecules allow to obtain a glimpse of the chemical reactivities of them. As the global hardness *η* represents the opposition of the molecular system to be deformed, it is clear that a large global hardness implies lower reactivity. Thus, MOL1 will be the most reactive, while MOL2 will be the least reactive of the five molecular systems considered here. The global electrophilicity *ω* represents a compromise between the behavior explicited by the electronegativy *χ* and that of the global hardness |eta and the results presented in Table 6 indicates that MOL1 will be display the most electrophilic character while for MOL5 it will be the opposite. In a similar way, MOL5 will be the molecule displaying the large nucleophilic character while MOL1 shows the minimum electrophilic character of all the systems. In accordance with this, MOL1 will top as having the large electrodonating character while MOL2 and MOL5 will be the ones with the smallest electroaccepting power.

## 4 Conclusion and future perspective

The currently approved medications for Alzheimer’s disease are centered on increasing cholinergic transmission by inhibiting ChE, however they only give modest improvements in memory and cognition. *Areca Catechu* L. leaf can be chosen as a prospective source of efficient ChE inhibitors, according to this research. Using a computational technique, this study sought to identify phytoconstituents that might bind to the crucial AD amyloid hypothesis targets while also testing the ability of the synthesized AC-HAp NPs to inhibit AChE. The produced material assumed a nanoflake particle, as evidenced by the analysis of AC-HAp NPs obtained by electron microscope examinations. Additionally, compared to AC extract, the AC-HAp NPs demonstrated improved anti-AChE and anti-BChE activity. According to docking scores and an examination of the interactions between the compounds, the majority of the compounds produced from *Areca Catechu* L. leaf extract had the capacity to bind to the chosen targets. The active compounds such as dehydronimodipine, *N*-acetylmuramic acid, 26,27-diethyl-1-alpha, 25-dihydroxy, *N*-acetylcilartatin, and nivalenol must be identified and their safety and bioavailability in animal models must be assessed further. Additional *in vivo* investigations of compounds would result in therapeutically efficient molecules for treating the issues related to AD.

## Data Availability

The original contributions presented in the study are included in the article/Supplementary Material, further inquiries can be directed to the corresponding authors.

## Abbreviations

AD, Alzheimer’s disease; AChE, Acetylcholinesterase; APP, Amyloid precursor protein; ATCI, Acetylthiocholine iodide; BChE, Butyrylcholinesterase; BSA, Bovine serum albumin; BTCI, Butyrylthiocholine iodide; CNS, Central nervous system; DTNB, 5, 5′-Dithio-bis-(2-nitrobenzoic acid); XRD, X-ray diffraction; SEM, scanning electron microscopy; TEM, transmission electron microscopy; SAED, selected area electron diffraction; FTIR, Fourier transform infrared spectroscopy; MDS, Molecular Dynamics Simulations; MD, Molecular Docking; RMSD, Root Mean Square Deviation; RMSF, Root Mean Square Fluctuation.

## References

[B1] Ankegowda V. M. Kollur S. P. Prasad S. K. Sushma P. Dhramashekara C. Jain A. S. (2020). Phyto-mediated synthesis of silver nanoparticles using Terminalia chebula fruit extract and evaluation of its cytotoxic and antimicrobial potential. Molecules 25, 5042. 10.3390/molecules25215042 PMC7662631 33143044

[B2] Atatreh N. Rawashdah S. A. Al Neyadi S. S. Abuhamdah S. M. Ghattas M. A. (2019). Discovery of new butyrylcholinesterase inhibitors via structure-based virtual screening. J. Enzyme Inhibition Med. Chem. 34 (1), 1373–1379. 10.1080/14756366.2019.1644329 PMC6711031 31347933

[B3] Avinash K. O. Sushma P. Chandan S. Gopenath T. S. Kiran M. N. Kanthesh B. M. (2021). *In silico* screened flavanoids of Glycyrrhiza glabra inhibit Cpla2 and Spla2 in lps stimulated macrophages. Bull. Env. Pharmacol. Life Sci. 10 (4), 14–24.

[B4] Chattaraj P. Chakraborty A. Giri S. (2009). Net electrophilicity. J. Phys. Chem. A 113, 10068–10074. 10.1021/jp904674x 19702288

[B5] Cramer C. J. (2004). Essentials of computational chemistry - theories and models. 2nd edn. Chichester, England: John Wiley & Sons.

[B6] Domingo L. R. Chamorro E. Perez P. (2008). Understanding the reactivity of captodative ethylenes in polar cycloaddition reactions. A theoretical study. J. Org. Chem. 73, 4615–4624. 10.1021/jo800572a 18484771

[B7] Domingo L. R. Perez P. (2011). The nucleophilicity N Index in organic chemistry. Org. Biomol. Chem. 9, 7168–7175. 10.1039/C1OB05856H 21842104

[B8] Domingo L. R. R´ıos-Gutie´rrez M. Pe´rez P. (2016). Applications of the conceptual density functional theory indices to organic chemistry reactivity. Molecules 21, 748. 10.3390/molecules21060748 PMC6273244 27294896

[B9] Domingo L. R. Sa´ez J. A. (2009). Understanding the mechanism of polar diels-alder reactions. Org. Biomol. Chem. 7, 3576–3583. 10.1039/B909611F 19675915

[B10] Ferreira-Vieira T. H. Guimaraes I. M. Silva F. R. Ribeiro F. M. (2016). Alzheimer's disease: Targeting the cholinergic system. Curr. Neuropharmacol. 14 (1), 101–115. 10.2174/1570159x13666150716165726 26813123 PMC4787279

[B11] Flores-Holguín N. Frau J. Glossman-Mitnik D. (2020a). A fast and simple evaluation of the chemical reactivity properties of the pristinamycin family of antimicrobial peptides. Chem. Phys. Lett. 739, 137021. 10.1016/j.cplett.2019.137021

[B12] Flores-Holguín N. Frau J. Glossman-Mitnik D. (2019a). Chemical-reactivity properties, drug likeness, and bioactivity scores of seragamides A–F anticancer marine peptides: Conceptual density functional theory viewpoint. Computation 7, 52. 10.3390/computation7030052

[B13] Flores-Holguín N. Frau J. Glossman-Mitnik D. (2019b). Computational peptidology assisted by conceptual density functional theory for the study of five new antifungal tripeptides. ACS Omega 4, 12555–12560. 10.1021/acsomega.9b01463 31460375 PMC6682088

[B14] Flores-Holguín N. Frau J. Glossman-Mitnik D. (2019c). Computational prediction of bioactivity scores and chemical reactivity properties of the Parasin I therapeutic peptide of marine origin through the calculation of global and local conceptual DFT descriptors. Theor. Chem. Acc. 138, 78. 10.1007/s00214-019-2469-3

[B15] Flores-Holguín N. Frau J. Glossman-Mitnik D. (2019d). “Conceptual DFT as a helpful chemoinformatics tool for the study of the clavanin family of antimicrobial marine peptides,” in Density functional theory calculations (Rijetia: IntechOpen), 1–11. 10.5772/intechopen.88657

[B16] Flores-Holguín N. Frau J. Glossman-Mitnik D. (2021). “Conceptual DFT as a helpful chemoinformatics tool for the study of the clavanin family of antimicrobial marine peptides,” in Density functional theory. Editors De Lazaro S. R. Da Silveira Lacerda L. H. Pontes Ribeiro R. A. (London, UK: IntechOpen), 57–67. chap. 3.

[B17] Flores-Holguín N. Frau J. Glossman-Mitnik D. (2020b). Conceptual DFT-based computational peptidology of marine natural compounds: Discodermins A-H. Molecules 25, 4158. 10.3390/molecules25184158 PMC7570683 32932850

[B18] Flores-Holguín N. Frau J. Glossman-Mitnik D. (2020c). Virtual screening of marine natural compounds by means of chemoinformatics and CDFT-based computational peptidology. Mar. Drugs 18, 478. 10.3390/md18090478 PMC7551818 32962305

[B19] Flores-Holguín N. Ortega-Castro J. Frau J. Glossman-Mitnik D. (2022). Conceptual DFT-based computational peptidology, pharmacokinetics study and ADMET report of the veraguamides A-G family of marine natural drugs. Mar. Drugs 20, 97. 10.3390/md20020097 35200627 PMC8874632

[B20] Frau J. Flores-Holguín N. Glossman-Mitnik D. (2018). Chemical reactivity properties, pKa values, AGEs inhibitor abilities and bioactivity scores of the mirabamides A–H peptides of marine origin studied by means of conceptual DFT. Mar. Drugs 16, 302–319. 10.3390/md16090302 PMC6163382 30154377

[B21] Frau J. Flores-Holguín N. Glossman-Mitnik D. (2019). Chemical reactivity theory and empirical bioactivity scores as computational peptidology alternative tools for the study of two anticancer peptides of marine origin. Molecules 24, 1115. 10.3390/molecules24061115 PMC6470772 30901820

[B22] Frau J. Glossman-Mitnik D. (2018a). Blue M2: An intermediate melanoidin studied via conceptual DFT. J. Mol. Model. 24, 138–213. 10.1007/s00894-018-3673-0 29855721

[B23] Frau J. Glossman-Mitnik D. (2018b). Chemical reactivity theory applied to the calculation of the local reactivity descriptors of a colored maillard reaction product. Chem. Sci. Int. J. 22, 1–14. 10.9734/CSJI/2018/41452

[B24] Frau J. Glossman-Mitnik D. (2018c). Computational study of the chemical reactivity of the blue-M1 intermediate melanoidin. Comput. Theor. Chem. 1134, 22–29. 10.1016/j.comptc.2018.04.018

[B25] Frau J. Glossman-Mitnik D. (2018e). Conceptual DFT study of the local chemical reactivity of the colored BISARG melanoidin and its protonated derivative. Front. Chem. 6, 136–139. 10.3389/fchem.2018.00136 29765937 PMC5938602

[B26] Frau J. Glossman-Mitnik D. (2018d). Conceptual DFT study of the local chemical reactivity of the dilysyldipyrrolones A and B intermediate melanoidins. Theor. Chem. Acc. 137, 67. 10.1007/s00214-018-2244-x

[B27] Frau J. Glossman-Mitnik D. (2018f). Molecular reactivity and absorption properties of melanoidin blue-G1 through conceptual DFT. Molecules 23, 559–615. 10.3390/molecules23030559 PMC6017537 29498665

[B28] Frisch M. J. Trucks G. W. Schlegel H. B. Scuseria G. E. Robb M. A. Cheeseman J. R. (2016). Gaussian 16 revision C.01. Wallingford CT: Gaussian Inc.

[B29] Gazquez J. Cedillo A. Vela A. (2007). Electrodonating and electroaccepting powers. J. Phys. Chem. A 111, 1966–1970. 10.1021/jp065459f 17305319

[B30] Geerlings P. Chamorro E. Chattaraj P. K. Proft F. D. Ga´zquez J. L. Liu S. (2020). Conceptual density functional theory: Status, prospects, issues. Theor. Chem. Acc. 139, 36. 10.1007/s00214-020-2546-7

[B31] Geerlings P. Proft F. D. Langenaeker W. (2003). Conceptual density functional theory. Chem. Rev. 103, 1793–1873. 10.1021/cr990029p 12744694

[B32] Heo J. H. Eom B. H. Ryu H. W. Kang M. G. Park J. E. Kim D. Y. (2020). Acetylcholinesterase and butyrylcholinesterase inhibitory activities of khellactone coumarin derivatives isolated from peucedanum japonicum thurnberg. Sci. Rep. 10, 21695. 10.1038/s41598-020-78782-5 33303801 PMC7730441

[B33] Jain A. S. Sushma P. Dharmashekar C. Beelagi M. S. Prasad S. K. Shivamallu C. (2021). *In silico* evaluation of flavonoids as effective antiviral agents on the spike glycoprotein of SARS-CoV-2. Saudi J. Biol. Sci. 28 (1), 1040–1051. 10.1016/j.sjbs.2020.11.049 33424398 PMC7783825

[B35] Jaramillo P. Domingo L. R. Chamorro E. Pe´rez P. (2008). Assessment of density functionals, semiempirical methods, and SCC-dftb for protonated creatinine geometries. Theochem 865, 68–73. 10.1016/j.theochem.2008.04.010 PMC2504755 19122761

[B36] Jensen F. (2007). Introduction to computational chemistry. 2nd edn. Chichester, England: John Wiley & Sons.

[B37] Kametani F. Hasegawa M. (2018). Reconsideration of amyloid hypothesis and tau hypothesis in Alzheimer's disease. Front. Neurosci. 12, 25. 10.3389/fnins.2018.00025 29440986 PMC5797629

[B38] Kanchanakungwankul S. Truhlar D. G. (2021). Examination of how well long-range-corrected density functionals satisfy the ionization energy theorem. J. Chem. Theory Comput. 17, 4823–4830. 10.1021/acs.jctc.1c00440 34319716

[B39] Kar R. Song J.-W. Hirao K. (2013). Long-range corrected functionals satisfy koopmans’ theorem: Calculation of correlation and relaxation energies. J. Comput. Chem. 34, 958–964. 10.1002/jcc.23222 23299544

[B40] Kim J. H. (2018). Genetics of Alzheimer's disease. Dement. Neurocogn. Disord. 17 (4), 131–136. 10.12779/dnd.2018.17.4.131 30906402 PMC6425887

[B41] Kollur S. P. Prasad S. K. Pradeep S. Veerapur R. Patil S. S. Amachawadi R. G. (2021). Luteolin-fabricated ZnO nanostructures showed PLK-1 mediated anti-breast cancer activity. Biomolecules 11 (3), 385. 10.3390/biom11030385 33807771 PMC7998981

[B42] Lewars E. (2003). Computational chemistry - introduction to the theory and applications of molecular and quantum mechanics. Dordrecht: Kluwer Academic Publishers.

[B43] Mahley R. W. Weisgraber K. H. Huang Y. (2006). Apolipoprotein E4: A causative factor and therapeutic target in neuropathology, including Alzheimer's disease. Proc. Natl. Acad. Sci. U. S. A. 103 (15), 5644–5651. 10.1073/pnas.0600549103 16567625 PMC1414631

[B44] Marenich A. V. Cramer C. J. Truhlar D. G. (2009). Universal solvation model based on solute electron density and on a continuum model of the solvent defined by the bulk dielectric constant and atomic surface tensions. J. Phys. Chem. B 113, 6378–6396. 10.1021/jp810292n 19366259

[B45] Mariwamy V. H. Kollur S. P. Shivananda B. Begum M. Shivamallu C. Dharmashekara C. (2022). N-((1H-Pyrrol-2-yl)methylene)-6-methoxypyridin-3-amine and its Co(II) and Cu(II) complexes as antimicrobial agents: Chemical preparation, *in vitro* antimicrobial evaluation, in silico analysis and computational and theoretical chemistry investigations. Molecules 27, 1436. 10.3390/molecules27041436 35209226 PMC8880514

[B46] Martorana A. Zaira E. Giacomo K. (2010). Beyond the cholinergic hypothesis : Do current drugs work in Alzheimer’s disease. CNS Neurosci. Ther. 16, 235–245. 10.1111/j.1755-5949.2010.00175.x 20560995 PMC6493875

[B47] Nistor M. Don M. Parekh M. SarsozaF. GoodusM. Lopez G. E. (2007). Alpha- and beta-secretase activity as a function of age and beta-amyloid in down syndrome and normal brain. Neurobiol. Aging 28 (10), 1493–1506. 10.1016/j.neurobiolaging.2006.06.023 16904243 PMC3375834

[B48] Peverati R. Truhlar D. G. (2012). Screened-exchange density functionals with broad accuracy for chemistry and solid-state physics. Phys. Chem. Chem. Phys. 14, 16187–16191. 10.1039/c2cp42576a 23132141

[B49] Pradeep S. Jain A. S. Dharmashekara C. Prasad S. K. Akshatha N. Pruthvish R. (2021). Synthesis, computational pharmacokinetics report, conceptual DFT-based calculations and anti-acetylcholinesterase activity of hydroxyapatite nanoparticles derived from acorus Calamus plant extract. Front. Chem. 9, 741037. 10.3389/fchem.2021.741037 34692640 PMC8529163

[B50] Prasad A. Govindaraju S. Sushma P. Jain A. S. Dharmasekar C. Prasad N. (2020). *Helicobacter pylori* infection: A bioinformatic approach. Int. J. Pharm. Sci. Res. 11, 5469–5483. 10.13040/IJPSR.0975-8232.11(11).1000-15

[B51] Prasad K. S. Pillai R. R. Ghimire M. P. Ray R. Richter M. Shivamallu C. (2020b). Indole moiety induced biological potency in pseudo-peptides derived from 2-amino-2-(1H-indole-2-yl) based acetamides: Chemical Synthesis, *in Vitro* Anticancer Activity and Theoretical Studies. J. Mol. Struct. 1217, 128445. 10.1016/j.molstruc.2020.128445

[B52] Prasad K. S. Pillai R. R. Shivamallu C. Prasad S. K. Jain A. S. Sushma P. (2020). Tumoricidal potential of novel amino-1, 10-phenanthroline derived imine ligands: Chemical preparation, structure, and biological investigations. Molecules 25, 2865. 10.3390/molecules25122865 PMC7356530 32580359

[B53] Prasad S. K. Pradeep S. Shimavallu C. Kollur S. P. Syed A. Marraiki N. (2021). Exploiting double exchange diels-alder cycloadditions for immobilization of peptide nucleic acids on gold nanoparticles. Front. Chem. 8, 4. 10.3389/fchem.2020.00004 PMC6989547 32039162

[B54] Srinivasa C. Kumar S. R. S. Pradeep S. Prasad S. K. Veerapur R. Ansari M. A. (2022). Eco-friendly synthesis of MnO2 nanorods using gmelina arborea fruit extract and its anticancer potency against MCF-7 breast cancer cell line. Int. J. Nanomedicine 17, 901–907. 10.2147/IJN.S335848 35250266 PMC8888196

[B55] Sushma P. Anisha S. J. Chandan D. Kollur S. P. Shashank K. P. Chandan S. (2020). Alzheimer's disease and herbal combination therapy: A comprehensive review. J. Alzheimers Dis. Rep. 4 (1), 417–429. 10.3233/ADR-200228 33283163 PMC7683102

[B56] Tsuneda T. Hirao K. (2014). Long-range correction for density functional theory. WIREs. Comput. Mol. Sci. 4, 375–390. 10.1002/wcms.1178

[B57] Tsuneda T. Song J.-W. Suzuki S. Hirao K. (2010). On koopmans’ theorem in density functional theory. J. Chem. Phys. 133, 174101. 10.1063/1.3491272 21054000

[B58] Uddin M. J. Russo D. Rahman M. Uddin S. B. Halim M. A. Zidorn C. (2021). Anticholinesterase activity of eight medicinal plant species: *In vitro* and in silico studies in the search for therapeutic agents against Alzheimer’s disease. Evidence-Based Complementary Altern. Med. 2021, 9995614. 10.1155/2021/9995614 PMC8260289 34257698

[B59] Uppar V. Chandrashekharappa S. Shivamallu C. Sushma P. Kollur S. P. Ortega-Castro J. (2021). Investigation of antifungal properties of synthetic dimethyl-4-bromo-1-(substituted benzoyl) pyrrolo[1, 2-a] quinoline-2, 3-dicarboxylates analogues: Molecular docking studies and conceptual DFT-based chemical reactivity descriptors and pharmacokinetics evaluation. Molecules 26 (9), 2722. 10.3390/molecules26092722 34066433 PMC8124935

[B60] Waring S. C. Rosenberg R. N. (2008). Genome-wide association studies in alzheimer disease. Arch. Neurol. 65 (3), 329–334. 10.1001/archneur.65.3.329 18332245

[B61] Weigend F. (2006). Accurate coulomb-fitting basis sets for H to Rn. Phys. Chem. Chem. Phys. 8, 1057–1065. 10.1039/b515623h 16633586

[B62] Weigend F. Ahlrichs R. (2005). Balanced basis sets of split valence, triple zeta valence and quadruple zeta valence quality for H to Rn: Design and assessment of accuracy. Phys. Chem. Chem. Phys. 7, 3297–3305. 10.1039/b508541a 16240044

[B63] Young D. (2001). Computational chemistry - a practical guide for applying techniques to real-world problems. New York: John Wiley & Sons.

