# Supplementary figures and images for: Anticholinesterase activity of Areca Catechu: In Vitro and in silico green synthesis approach in search for therapeutic agents against Alzheimer’s disease
Source: Front Pharmacol. 2022 Nov 4;13:1044248. doi: 10.3389/fphar.2022.1044248 (PMC9672481; doi:10.3389/fphar.2022.1044248)

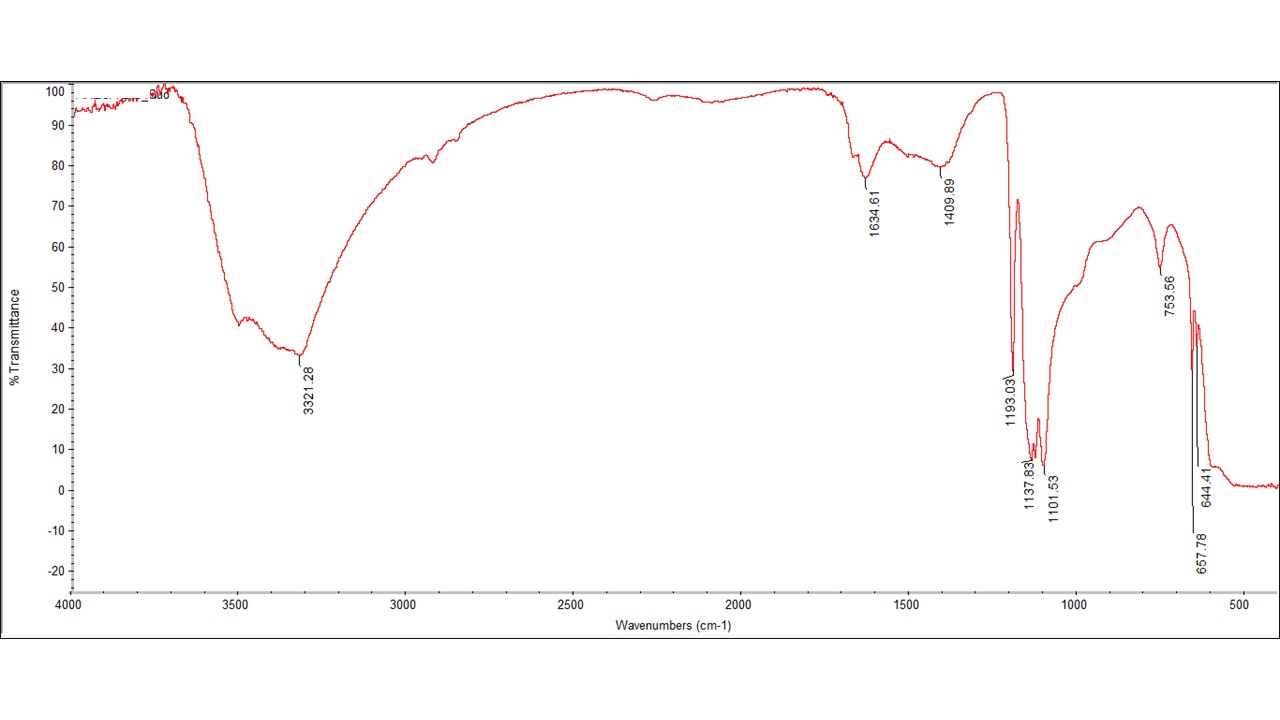

Supplement: Supplementary file 1 [file Image1.JPEG]
